# Mature oocyte dysmorphisms may be associated with progesterone levels, mitochondrial DNA content, and vitality in luteal granulosa cells

**DOI:** 10.1007/s10815-024-03053-5

**Published:** 2024-02-16

**Authors:** Georges Raad, Judy Tanios, Munevver Serdarogullari, Marwa Bazzi, Youmna Mourad, Joseph Azoury, Zalihe Yarkiner, Georgios Liperis, Fadi Fakih, Chadi Fakih

**Affiliations:** 1 Al Hadi Laboratory and Medical Center, Beirut, Lebanon; 2 Faculty of Medicine and Medical Sciences, Holy Spirit University of Kaslik (USEK), Jounieh, Lebanon; 3 IVF Lebanon, Embryology, Hazmieh Lebanon; 4 Department of Histology and Embryology, Faculty of Medicine, Cyprus International University, Northern Cyprus Via Mersin 10, Mersin, Turkey; 5 Azoury IVF Clinic, ObGyn and Infertility, Beirut, Lebanon; 6 Faculty of Arts and Sciences-Department of Basic Sciences and Humanities, Cyprus International University, Northern Cyprus Via Mersin 10, Mersin, Turkey; 7 Westmead Fertility Centre, Institute of Reproductive Medicine, University of Sydney, Westmead, NSW Australia; 8 Faculty of Medicine, Lebanese University, Beirut, Lebanon

**Keywords:** Oocytes, Mitochondria, Granulosa cells, Estradiol, Progesterone

## Abstract

**Purpose:**

To identify whether follicular environment parameters are associated with mature oocyte quality, embryological and clinical outcomes.

**Methods:**

This retrospective study examined 303 mature oocytes from 51 infertile women undergoing ICSI cycles between May 2018 and June 2021. Exclusion criteria consisted of advanced maternal age (> 36 years old), premature ovarian failure, obesity in women, or use of frozen gametes. Luteal granulosa cells (LGCs) were analyzed for mitochondrial DNA/genomic (g) DNA ratio and vitality. The relationships between hormone levels in the follicular fluid and oocyte features were assessed. Quantitative morphometric measurements of mature oocytes were assessed, and the association of LGC parameters and oocyte features on live birth rate after single embryo transfer was examined.

**Results:**

Results indicated an inverse correlation between the mtDNA/gDNA ratio of LGCs and the size of polar body I (PBI). A 4.0% decrease in PBI size was observed with each one-unit increase in the ratio (*p* = 0.04). Furthermore, a 1% increase in LGC vitality was linked to a 1.3% decrease in fragmented PBI (*p* = 0.03), and a 1 ng/mL increase in progesterone levels was associated with a 0.1% rise in oocytes with small inclusions (*p* = 0.015). Associations were drawn among LGC characteristics, perivitelline space (PVS) debris, cytoplasmic inclusions, PBI integrity, and progesterone levels. Certain dysmorphisms in mature oocytes were associated with embryo morphokinetics; however, live birth rates were not associated with follicular parameters and oocyte quality characteristics.

**Conclusion:**

Follicular markers may be associated with mature oocyte quality features.

**Supplementary Information:**

The online version contains supplementary material available at 10.1007/s10815-024-03053-5.

## Introduction

The clinical pregnancy rates following intracytoplasmic sperm injection (ICSI) continue to be around 30%, indicating that there is still room for advancement in this area [1, 2]. Several systematic reviews and meta-analyses indicated that the success rates of ICSI can vary based on the quality of the oocyte used (e.g., abnormal first polar body size, cytoplasmic vacuoles, central cytoplasmic granularity) [3–5].

The quality of an oocyte is impacted by its own genome as well as the microenvironment in which it matures [6]. Particularly, the oocyte, granulosa cells, and theca cells form a complex and dynamic unit known as the ovarian follicle [7, 8]. During the formation of the antral follicle, granulosa cells differentiate into cumulus cells and mural granulosa cells under the influence of gonadotropins and oocyte-secreted factors. The bidirectional communication of the oocyte and cumulus cells regulates oocyte maturation and metabolism [7, 8]. Cumulus cells play a crucial role in supplying the oocyte with vital small-molecule compounds necessary for development, including essential nutrients for energy production and factors governing meiotic control. In turn, oocytes release growth factors that regulate various functions of cumulus cells, such as proliferation, apoptosis prevention, and steroidogenesis. In addition, these cells exhibit distinct carbohydrate metabolic profiles, with cumulus cells metabolizing significantly more glucose than oocytes due to the presence of high-affinity glucose transporters and enriched glycolytic enzymes. Conversely, oocytes prioritize pyruvate metabolism through the tricarboxylic acid cycle and oxidative phosphorylation, consuming notably higher oxygen levels [9] [10]. Therefore, cumulus cells play a pivotal role in furnishing the oocyte with substrates crucial for the tricarboxylic acid cycle and oxidative phosphorylation during oocyte maturation [9] [10]. The cumulus cells also produce hyaluronic acid (HA) leading to extracellular matrix expansion, which helps with ovulation and increases the chances of fertilization [7, 8, 11]. Moreover, mural granulosa cells regulate the COC expansion and oocyte maturation by secreting paracrine factors [12]. In parallel, mural granulosa cells produce estrogen during the follicular phase, influencing oocyte quality by modulating intracellular calcium homeostasis [8, 13, 14]. After the LH surge, mural granulosa cells enlarge to form luteinized cells and produce progesterone, regulating oocyte maturation [8, 15]. Estrogen and progesterone are both synthesized in the mitochondria, and these sex steroid hormones may play a role in regulating mitochondrial activity [16–20].

Given the crucial role of luteinized granulosa cells (LGCs) in oocyte development and the maintenance of pregnancy, they are frequently utilized as a model for studying various aspects of ovarian function. Furthermore, LGCs are deemed valuable tools in reproductive biology studies, thanks to their easy accessibility during assisted reproduction techniques [83]. Various studies showed that mitochondrial changes in LGCs can be influenced by various factors, including reproductive aging, polycystic ovary syndrome (PCOS), and the presence of ovarian endometrioma [21–23]. These changes can result in decreased mitochondrial membrane potential, reduced adenosine triphosphate (ATP) synthesis, and increased oxidative stress, potentially affecting fertility through decreased steroidogenesis and impairing oocyte quality [21, 22]. However, the relationships between changes in LGCs quality and oocyte quality, such as the presence of cytoplasmic dysmorphisms and extracytoplasmic dysmorphisms, remain unexplored and not fully elucidated.

Nowadays, the shift towards transferring a single embryo to reduce the risk of multiple pregnancies has heightened the need for more effective methods of selecting oocytes and embryos with the highest chance of leading to a successful pregnancy, rather than relying solely on morphological evaluation [24]. However, invasive methods for analyzing oocytes are not practical as they hinder their use in assisted reproduction. Hence, finding objective and non-invasive ways to evaluate oocyte quality is crucial [25]. Time-lapse screening of oocyte quality and embryo development is an advanced version of the traditional method of morphological evaluation as it provides a continuous analysis of the early embryo development [26, 27]. However, its impact on improving pregnancy rates is still controversial and not widely accepted [28, 29]. In this regard, molecular markers can serve as additional tools to support embryo selection based on morphokinetics and morphodynamic features [24, 30].

Identification of markers in the follicular environment that can positively or negatively affect the acquisition of cytoplasmic and molecular oocyte maturity can provide insights into patient embryological and clinical outcomes. This information can help us understand the reasons behind observed dysmorphisms in cohorts of oocytes collected from patients and their subsequent compromised embryo development. Understanding the mechanisms and effects behind oocyte dysmorphisms can be incorporated into patient counseling and managing expectations. Thus, the aim of this study is to combine a multitude of assessments originating from the follicular environment, including the morphology of LGCs, vitality of LGCs, mitochondrial (mt)DNA/genomic (g)DNA ratio in the LGCs, as well as estradiol and progesterone levels in the follicular fluid, and link them to oocyte features. Furthermore, we aim to relate the association of different oocyte features with embryo morphokinetics and clinical outcomes.

## Materials and methods

### Study design and ethical approval

This is a multicenter retrospective study conducted on couples (*n* = 51) who received ICSI treatment at Azoury IVF Clinic, Hazmieh; Mount Lebanon Hospital, Hazmieh; and Al Hadi Laboratory and Medical Center, Beirut, Lebanon between May 2018 and June 2021. The study received an ethical approval from Mount Lebanon Hospital, Hazmieh, Lebanon (OBS-2018–002). All included patients signed informed consent forms for the use of their data for research purposes.

### Participants

Exclusion criteria consisted of advanced maternal age (> 36 years old) at the time of oocyte pickup (OPU), use of frozen gametes, patients with poor ovarian response following stimulation (< 3 cumulus-oocyte complexes obtained), couples undergoing ICSI with testicular biopsy/epididymal sperm aspiration, and cases where a high spermatic DNA fragmentation index (> 30% as determined by the sperm chromatin dispersion test) was identified [31]. Cases of severe male factor infertility (< 5 mil/ml sperm) were also excluded from the study, as compromised sperm quality characteristics may influence early embryo development [31, 32]. Patients with no good quality embryos available for transfer on day 4, as assessed by evidenced initiation of compaction were also excluded.

### Ovarian stimulation, oocyte pick up, ICSI, embryo culture

Ovarian stimulation was performed using recombinant follicle-stimulating hormone (FSH) (Gonal-f, Merck Serono, Lebanon). A GnRH antagonist (0.25 mg/day, Cetrotide, 0.25 mg, Merck Serono, Lebanon) was administered to prevent a premature luteinizing hormone (LH) surge. Once at least three follicles reached 16 mm in diameter, ovulation was triggered with human chorionic gonadotropin (hCG) (Ovitrelle, Merck Serono, Lebanon) [32]. Thirty-four to 36 h after ovulation induction, cumulus-oocyte complexes (COCs) were retrieved and incubated in culture medium covered with oil (Life Global, Ibra Haddad, Lebanon) at 37 °C, and under 5% O_2_, and 5% CO_2_ for 2–3 h [31].

On the same day of OPU, fresh semen samples were collected from male partners and analyzed according to the World Health Organization 2010 guidelines (WHO, 2010). Semen samples were processed by density gradient centrifugation using two-layered density gradient (80–40 gradient layers, COOK Medical, EMEC, Lebanon). The sperm pellet was washed with sperm medium (COOK Medical, EMEC, Lebanon) prior to resuspension in 0.5 ml of sperm medium (COOK Medical, EMEC, Lebanon) until ICSI. The prepared sperm samples were placed in a 10% polyvinylpyrrolidone (PVP) (Irvine Scientific, IV laboratory, Lebanon) solution during ICSI procedure. ICSI was employed as the fertilization method for all the included couples, where each mature oocyte was injected with an immobilized spermatozoon in HEPES medium (Life Global, Ibra Haddad, Lebanon) under lite oil (Life Global, Ibra Haddad, Lebanon). The injected oocytes were transferred to a 12-well pre-equilibrated EmbryoSlide (Vitrolife, Biofert, Lebanon) containing global® total® LP (CooperSurgical®) medium covered with lite oil (Life Global, Ibra Haddad, Lebanon). The EmbryoSlides were then placed in an Embryoscope chamber and cultured for 4 days (37 °C, 5% O_2_, 5% CO_2_) [33]. Embryos were graded on day 4 using embryo viewer and for each patient, a single embryo transfer (SET) was performed on day 4. Embryos deriving from normal morphology oocytes were preferentially chosen for ET. For luteal support, daily vaginal progesterone (Crinone 8%, Merck Serono) was used [32, 34].

## Data sources

### Estradiol and progesterone levels

Follicular fluid, obtained through transvaginal ultrasound-guided follicular puncture during routine oocyte harvest, was pooled from each patient. The collected follicular fluid free of COCs underwent visual inspection for the presence of red blood cells (RBCs). RBCs have the ability to bind hormones, such as estrogen, potentially influencing the accuracy of hormonal level measurements. Only follicular fluid samples free of visible blood contamination were included for analysis. Subsequently, the follicular fluid was purified through centrifugation at 448 g for 10 min in order to minimize RBC contamination in the follicular fluid [35–37]. The levels of estradiol and progesterone were measured et al.-Hadi Laboratory and Medical Center using commercially available kits and following manufacturer instructions (Cobas e411; Roche Diagnostics) [38].

### LGC purification and assessment of their viability and morphology

The luteal granulosa cells (LGC) aggregates were collected from the follicular fluid free of cumulus-oocyte complexes using a stereomicroscope (Olympus SZ61). For each case, the collected cells were pooled and transferred into a conical tube consisting of two layers of culture media with distinct densities (80–40 gradient layers, COOK Medical, EMEC, Lebanon), followed by centrifugation at 448 g for 10 min. A ring-like layer formed at the interface of the density gradient was obtained and transferred to a round tube and mixed with 1 ml of HTF (Human Tubal Fluid, Quinn’s Advantage Medium with HEPES, Ibra Haddad, Lebanon) before centrifugation for an additional 10 min at 161 g. The resulting pellet was suspended in 1-ml HTF [39].

To assess cell viability, a mixture of Trypan blue (0.4%) exclusion dye and cell suspension was created (v/v), incubated at room temperature for 3 min, and then counted on a hemocytometer. The cell viability was calculated by dividing the number of live cells by the number of dead cells [39].

The morphology of the LGCs was also evaluated. A thin smear slide was prepared from the cell suspensions and stained using Wright-Giemsa. The slides were then examined by a clinical pathologist to identify the percentage of LGCs with normal morphology [39]. For the assessment, LGCs exhibiting a prominent dark-stained nucleus, a paler cytoplasm with a foamy appearance, and an intact cell structure without any cytoplasmic shrinkage were recorded as cytologically normal [39].

### Mitochondrial DNA to genomic DNA ratio in LGCs

A commercial kit (Genomic DNA from tissue kit, Nucleospin, Meato, Lebanon) was used to extract DNA from the LGCs. Briefly, 10^6^ cells were re-suspended in a mixture of 200-µl lysis buffer T1, 25-µl proteinase K, and 200-µl lysis buffer B3 (containing guanidine hydrochloride a chaotropic salt), which was then vortexed and incubated at 70 °C for 15 min. After adding 200 µl of 96% ethanol and vortexing the solution again, it was loaded onto a nucleospin tissue column. The DNA was eluted with 100-µl BE buffer after being washed with 500 µl wash buffer BW (containing guanidine hydrochloride a chaotropic salt) and 600 µl wash buffer B5 (wash buffer with 96–100% ethanol). Subsequently, DNA was eluted from the column using 100 µl of elution buffer (BE buffer). The extracted DNA was analyzed using electrophoresis on a 0.7% agarose gel (100 mV, 30 min) using a Bio-Rad Laboratories apparatus. As per the guidelines provided by the manufacturer in the user manual (Thermo Fisher Scientific), DNA amplification and library preparation were executed employing the Ion Single Seq 96 Kit from the Ion ReproSeq PGS Kit, along with Ion 530 Chips. Subsequently, the libraries underwent sequencing through next-generation sequencing (NGS) using an Ion S5 sequencer (Thermo Fisher Scientific). The data analysis was conducted utilizing the Reproseq PGS w1.1 workflow on Ion Reporter, applying standard parameters. The ratio of mitochondrial to genomic DNA (mtDNA/gDNA) was calculated using the Ion Torrent system (Thermo Fisher, Lebanon) as previously described [40, 41]. Specifically, the number of mtDNA reads was divided by the number of reads attributed to the nuclear genome, resulting in a quantitative assessment of mtDNA content per cell [40].

### Assessment of oocyte quality parameters

**Fig. 1:**
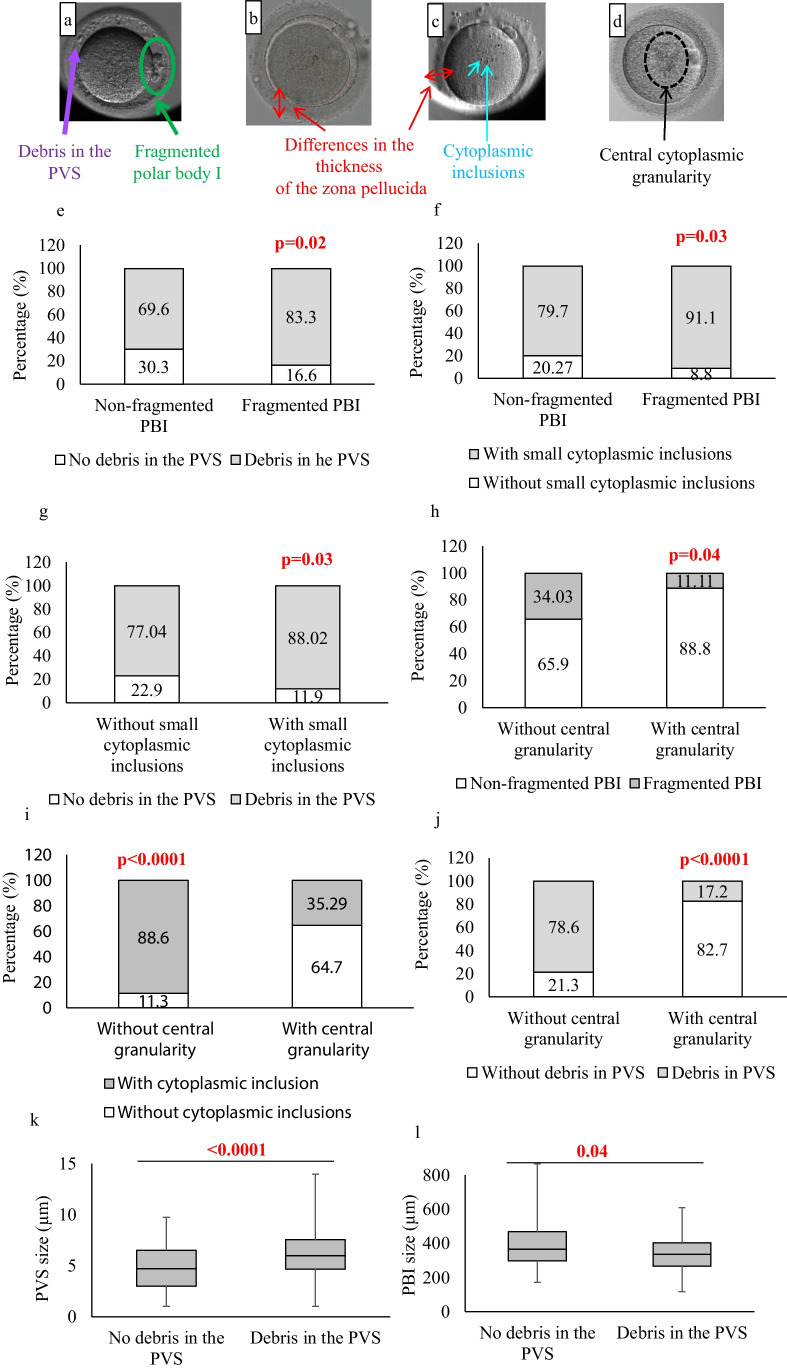
Dysmorphic phenotypes in MII oocytes and their relationships (a–d): Representative images of dysmorphic MII oocyte phenotypes. (a) Fragmented polar body I (green circle) and debris in the perivitelline space (purple arrow). (b, c) Variation in the thickness of the zona pellucida (red arrow). (c) MII oocyte with cytoplasmic inclusions (aqua arrow). (d) MII oocyte displaying central cytoplasmic granularity (black circle). (e–j): Stacked column charts showing the relationships between mature oocyte dysmorphisms (categorical variables). (k, l): Boxplots showing relationships between mature oocyte dysmorphisms (continuous variables). (k) Boxplots comparing the PVS size (µm) in mature oocytes with and without debris in the PVS (continuous variables). (l) Boxplots comparing the PBI size (µm) in mature oocytes with and without debris in the PVS (continuous variables). Statistical significance was considered when *p* < 0.05

Immediately after the ICSI procedure, oocytes (*n* = 303) were transferred to an Embryoslide in the Embryoscope chambers connected to the Embryo Viewer software. The software’s imaging system was configured to take high-definition images of each oocyte/embryo from seven different focal planes, at 15-min intervals. In the context of identifying oocyte features, analysis of all seven available imaging planes allowed comprehensive visualization of features that may not be discernible in each individual plane. For morphometric analysis of oocytes, all seven distinct planes were examined, and the plane that best represented the oocyte was selected for the follow-up analysis. Oocyte quality parameters were objectively measured and recorded by two embryologists who were blinded to the quality of the luteal granulosa cells (Fig. 1). In this study, the criteria for oocyte morphology were classified following the guidelines of the Istanbul Consensus Workshop on embryo assessment. The categorization included the expansion of the oocyte–corona–cumulus complex, extracytoplasmic parameters (such as variations in first polar body (PBI) size and fragmentation, perivitelline space dimensions and debris, and zona pellucida size), and intracytoplasmic anomalies (including small cytoplasmic inclusions, central cytoplasmic granulation, and cytoplasmic vacuoles measuring < 14 µm) [45, 46]. In this context, quantitative morphometric measurements included the zona pellucida thickness, the size of the PBI, size of the perivitelline space (PVS), and the size of the vacuoles [42] (Fig. 1(a–d)). The thickness of the zona pellucida and the size of the PVS were measured by drawing several lines across each and calculating the average thickness and size, respectively, in micrometers [43]. The diameters of the PBI and the vacuoles were measured using the elliptical measurement tool [44]. Additionally, the presence of intracytoplasmic anomalies, such as PBI fragmentation; the presence of debris in the perivitelline space (PVS); cytoplasmic inclusions like refractile bodies or lipofuscin bodies; and central cytoplasmic granularity (also referred to as highly concentrated central granularity) were individually assessed for each mature oocyte using the Embryo Viewer software [6, 46] (Fig. 1). Only oocytes with large vacuoles (> 14 μm) were excluded from the study [45]. The presence of each anomaly was examined, and the percentage of each anomaly among the mature oocytes per patient was also calculated. The cumulus-corona mass, which is typically described as expanded, “cloud-like” or fluffy due to the secretion of hyaluronic acid by the cumulus cells, was also evaluated [6, 46]. The percentage of the expanded cumulus oocyte complexes per patient was also assessed.

### Calculation of maturation and fertilization rates

Oocyte maturation rate was evaluated by dividing the number of metaphase II oocytes by the number of COC retrieved per patient. An oocyte was categorized as mature when the first polar body was extruded and visible in the PVS [6, 46, 47].

The fertilization rate was calculated from the number of fertilized oocytes divided by the total number of inseminated oocytes. Normally fertilized oocytes were defined as those with two polar bodies and two pronuclei [6, 46].

### Embryo morphokinetic parameters assessment

The embryo morphokinetic parameters were evaluated by following established guidelines from previous publications [48, 49]. The parameters assessed included the time for pronuclear fading (tPNf), the time for division into two cells (t2), and the subsequent division times for three cells (t3), four cells (t4), five cells (t5), and eight cells (t8). Additionally, the time for start compaction and full compaction were measured. All of these morphokinetic events were measured in hours post-insemination (hpi), with the end of ICSI considered as the time of insemination. Regarding embryo morphokinetics, the utilization of various focal planes facilitated clearer identification of the precise time points for each morphokinetic event.

### Sample size calculation

The objective of this study was to examine several factors from the follicular environment such as the impact of mtDNA/gDNA ratio in LGCs, estradiol levels, progesterone levels, LGC morphology, and LGC viability on various dependent variables (oocyte quality parameters). Given that the dependent variables involved repeated measures, the analysis was performed using a generalized linear mixed model approach. The G-Power 3.1 software program was used to calculate the necessary sample size for this study, taking into account five independent predictors (mtDNA/gDNA ratio in LGCs, estradiol levels, progesterone levels, LGC morphology, and LGC viability). The parameters for the sample size calculation were set as a power of 0.8, alpha level of 0.05, and an effect size *f*^2^ of 0.15. According to a one-sample *t* test family, the program calculated the desired sample size to be 43. To account for a possible 10% dropout rate, the minimum sample size was determined to be 48.

### Statistical analysis

The data was analyzed using SPSS version 25, where a *p* value of less than 0.05 was considered statistically significant. The Kolmogorov–Smirnov test was used to test normality of continuous parameters. The data for normally distributed continuous parameters was presented as mean ± standard deviation (SD), while non-normally distributed continuous parameters were shown as median (minimum–maximum or interquartile range) values. Additionally, categorical variables were presented as number (percentage (%). Student’s *t* tests and Mann–Whitney *U* tests (for variables that did not follow a normal distribution) were conducted for continuous data, and chi-square tests were used for categorical data. Pearson and Spearman correlation tests were employed to assess the relationship between the variables [50, 51].

### Generalized linear mixed models

The primary focus of this study was to investigate the impact of distinctive characteristics of LGCs and hormone levels within the follicular fluid on the extra- and intra-cytoplasmic dysmorphisms of mature human oocytes. The assessed dysmorphisms included the size of the PVS (µm), the percentage of oocytes showing debris within the PVS, the size of the PBI (µm), the percentage of oocytes exhibiting fragmented PBI, and the percentages of oocytes displaying small vacuoles, small inclusions, and central granularity. Thus, seven distinct generalized linear mixed models (GLMMs) were implemented, with each model addressing one of the identified extra- or intra-cytoplasmic dysmorphisms of mature human oocytes separately. In the GLMM models, the target was related to the factors and covariates through a specified link function. The log link function was employed for continuous variables. The distribution of each oocyte quality parameter was used to determine which probability distribution should be employed in the model. The gamma distribution was used when the target contained only positive values and was skewed towards larger values. It is worth mentioning that among the observed measurements, specific characteristics, such as the percentages of oocytes with debris in the PVS, fragmented PBI, small cytoplasmic vacuoles, small cytoplasmic inclusions, and central cytoplasmic granularity, were measured only once. As a result, the dataset comprised 51 individual measurements, each obtained from a different patient. In contrast, other variables, namely, PVS size (µm) and PBI size (µm), involved 12 repeated measurements per patient, resulting in a total dataset of 612 measurements. Consequently, GLMMs were employed to account for the repeated-measure structure of the data, leveraging the comprehensive 612 measurements for these specific variables.

### Categorical principal component analysis

The categorical principal component analysis (CATPCA), a statistical technique employed to discern patterns among categorical variables, was used. Unlike its counterpart, principal component analysis (PCA), which deals with continuous variables, CATPCA is tailored to handle categorical data. It involves converting the categorical variables into a set of continuous variables through various encoding methods. Subsequently, it applies the principles of PCA to reveal the principal components that account for the most variance within the data. This approach aids in uncovering intricate relationships within the categorical variables in this dataset, facilitating insightful interpretations and meaningful analyses [52] [53].

## Results

### Population characteristics

Demographic details, sperm parameters, LGCs parameters, and progesterone and estradiol levels are displayed in Table 1. The mean age of the women included in this study was 29.98 ± 5.54 years, with a median age of their male partners 36 years (25–54) (Table 1). The mean BMI for women and men was 23.33 ± 3.24 kg/m^2^ and 28.75 ± 4.14 kg/m^2^, respectively (Table 1).

**Table 1:** Population characteristics

		Values
Women	Age (years)	29.98 ± 5.54
	Body mass index (BMI) (kg/m^2^)	23.33 ± 3.24
	PCOS (ratio (%))	5/51 (9.8)
	Endometrioma (ratio (%))	1/51 (1.96)
	Polypectomy (ratio (%))	2/51 (3.92)
	Primary infertility (ratio (%))	37/51 (72.5)
	Secondary infertility (ratio (%))	14/51 (27.4)
	Previous miscarriage(s)	
	0 (ratio (%))	30/51 (58.8)
	1 (ratio (%))	9/51 (17.6)
	2 (ratio (%))	4/51 (7.8)
	3 (ratio (%))	5/51 (9.8)
	4 (ratio (%))	2/51 (3.92)
	Previous ICSI attempts	
	0 (ratio (%))	29/51 (56.8)
	1 (ratio (%))	13/51 (25.4)
	2 (ratio (%))	3/51 (5.88)
	3 (ratio (%))	2/51 (3.92)
	4 and more (ratio (%))	3/51 (5.88)
	Family history of infertility (ratio (%))	14/51 (27.45)
	Current smoker (ratio (%))	24/51 (47)
	Current alcohol consumption (ratio (%))	3/51 (5.8)
Men	Age (men) (years)	36 (25–54)
	Body mass index (BMI) (men) (kg/m^2^)	28.75 ± 4.1
	Varicocelectomy (%)	3/51 (5.88)
	Orchidopexy (%)	4/51(7.84)
	Inguinal hernia surgery (%)	2/51(3.92)
	Current smoker (ratio (%))	37/51 (60.7)
	Current alcohol consumption (ratio (%))	15/51(29.4)
	Sperm concentration × 10^6^/ml	20 (0.1–76)
	Progressive motility (%)	25 (2–40)
	Non-progressive motility (%)	30 (5–40)
	Non-motile (%)	45 (30–90)
Luteal granulosa cells characteristics, estradiol level, and progesterone level in the follicular fluid	mtDNA/gDNA ratio in LGCs (× 10^–4^)	4 (1–9)
	LGCs normal morphology (%)	81 (53–96)
	LGCs vitality (%)	80 (33–100)
	Estradiol (× 10^4^) (pg/ml)	154.506 ± 62.33
	Progesterone (× 10^4^) (ng/ml)	4.37 ± 1.7
	Number of COC recovered	11 (3–28)
Oocyte quality	Percentage of expanded COC (%)	64.29 (0–100)
	Zona pellucida thickness (µm)	16.2 (3–24)
	Percentage of oocytes with debris in PVS	82.86 (9.09–100)
	PVS size (µm)	5.75 (1–14)
	First polar body (PBI) size (µm)	354.4 ± 117.17
	Percentage of oocytes with fragmented PBI (%)	33.33 (0–100)
	Maturation rate (%)	75 (0–100)
	Percentage of oocytes with small vacuoles (%)	0 (0–83.33)
	Percentage of oocytes with small inclusions (%)	100 (0–100)
	Percentage of oocytes with central granularity (%)	0 (0–66.67)
	Percentage of oocytes with smooth endoplasmic reticulum clusters (%)	0
Fertilization rate (%)		70.19 ± 26.77

Data expressed as mean ± standard deviation for normally distributed continuous variable, as median (minimum–maximum) for non-normally distributed variable, and as percentage for categorical variable

*PCOS* polycystic ovary syndrome, *mtDNA* mitochondrial DNA, *LGCs* luteal granulosa cells, *COC* cumulus-oocyte complex, *PVS* perivitelline space

### Incidence of oocyte dysmorphisms and their interrelationships

The percentage of expanded cumulus-oocyte complexes (COC) was 64.29 (0–100). With regard to the extracellular parameters, the median thickness of the zona pellucida was 16.2 μm (3–24 μm), the median size of PVS was 5.75 μm (1–14 μm), and the average size of the PBI was 354.4 ± 117.17 μm. Furthermore, the percentages of oocytes with fragmented PBI or debris in the PVS were 33.33% (0–100%) and 82.86% (9.09–100%), respectively. In terms of the intracellular parameters, the percentages of oocytes with small cytoplasmic vacuoles, small cytoplasmic inclusions, and central cytoplasmic granularity were 0% (0–83.3%), 100% (0–100%), and 0% (0–66.67%), respectively (Table 1). None of the oocytes included in this study presented smooth endoplasmic reticulum clusters (Table 1).

A chi-square test of independence was conducted to investigate the relationship between different mature oocyte dysmorphisms. The analysis revealed that the presence of debris in the PVS was more likely to take place in oocytes with fragmented PBI (*p* = 0.02) (Fig. 1(e)). Furthermore, a significant association was observed between oocytes with fragmented PBI and the presence of small cytoplasmic inclusions (*p* = 0.03) (Fig. 1(f)). Likewise, a significant connection was detected between the existence of small cytoplasmic inclusions and the presence of debris in the PVS (*p* = 0.03) (Fig. 1(g)).

In contrast, oocytes displaying central cytoplasmic granularity had a lower occurrence of fragmented PBI (*p* = 0.04) (Fig. 1(h)). Conversely, oocytes without central cytoplasmic granularity exhibited a notably higher percentage of small cytoplasmic inclusions (*p* < 0.0001) (Fig. 1(i)). Additionally, a small proportion of oocytes had debris in the PVS when central cytoplasmic granularity was present (*p* < 0.0001) (Fig. 1(j)). Simultaneously, the size of the PVS was found to be larger in oocytes with debris in the PVS (*p* < 0.0001) (Fig. 1(k)). Furthermore, the size of PBI was smaller in oocytes with debris in the PVS (*p* = 0.04) (Fig. 1(l)). No statistically significant associations were found between the presence of small cytoplasmic vacuoles with other features such as PBI integrity, presence of debris in the PVS, small cytoplasmic inclusions, or central cytoplasmic granularity.

### Exploring the relation between luteal granulosa cells characteristics, estradiol level, and progesterone level in the follicular fluid

**Fig. 2:**
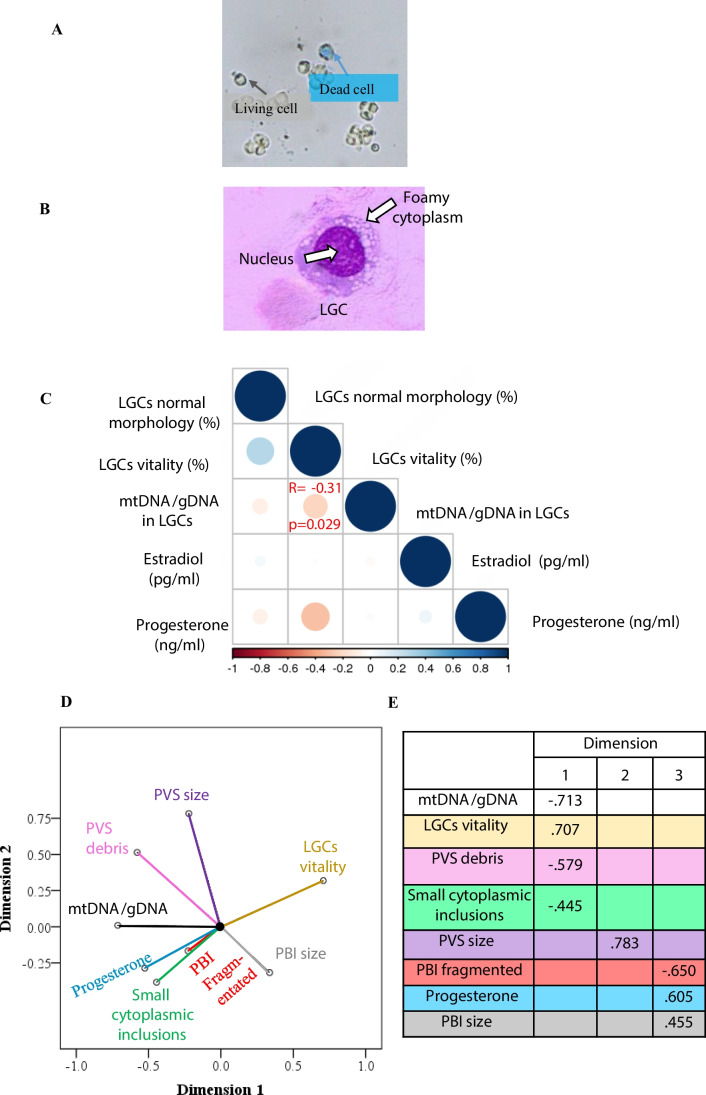
Characterization of luteal granulosa cells and their association with oocyte dysmorphisms. **a** Viable and dead cells were stained with trypan blue, with dead cells appearing blue and viable cells appearing transparent. **b** Granulosa cells exhibit a central nucleus (purple) and foamy cytoplasm (light purple). **c** Correlation matrix plot of all luteal granulosa cell (LGC) variables assessed. The legend color represents the correlation coefficients, with darker shades indicating stronger correlations. Statistical significance was considered when *p* < 0.05. **d**, **e** Categorical principal component analysis (CATPCA) biplot showing the component loadings of the most explanatory variables in LGCs and oocyte dysmorphisms

To assess if there was a relationship between LGCs characteristics (Fig. 2A, B), estradiol level, and progesterone level in the follicular fluid, Spearman’s correlation coefficient test was performed (Fig. 2C). The result of this test showed that there was only a significant negative correlation between mtDNA/gDNA ratio in luteal granulosa cells and percentage of luteal granulosa cells vitality (*R* =  − 0.313, *p* = 0.029).

*The characteristics of luteal granulosa cells, along with estradiol and progesterone levels in the follicular fluid, may influence the extra- and intracytoplasmic dysmorphisms of mature human oocytes.*

#### PVS size and debris presence

The results of the GLMM indicated that the size of the PVS (µm) and the presence of debris in the PVS were not influenced by the evaluated independent variables (Table 2), including mtDNA/gDNA ratio in luteal granulosa cells, estradiol level, progesterone level, percentage of luteal granulosa cells with normal morphology, and vitality of luteal granulosa cells.

**Table 2:** Luteal granulosa cell characteristics and hormone levels in follicular fluid may be associated to extra- and intra-cytoplasmic dysmorphisms in mature human oocytes

								95% confidence interval	
Dependent variable	Independent variables	Coefficient	Standard error			*t*-value	*p* value	Lower	Upper
The size of the perivitelline space (µm)	Intercept	1.463	0.447			3.275	0.001	0.583	2.342
	mtDNA/gDNA ratio	0.033	0.025			1.328	0.185	− 0.016	0.083
	Estradiol levels	− 0.0001	0.0001			− 1.089	0.277	− 0.0001	0.0001
	Progesterone levels	0.0001	0.0001			0.459	0.647	− 0.0001	0.0001
	LGCs normal morphology	0.002	0.004			0.692	0.490	− 0.004	0.009
	LGCs vitality	0.001	0.004			0.281	0.779	− 0.007	0.009
	*Probability distribution: normal, link function: log*								
% of oocytes with debris in the perivitelline space	Intercept	113.217	39.947			2.834	0.007	32.349	194.085
	mtDNA/gDNA ratio	2.302	2.486			0.926	0.360	− 2.731	7.336
	Estradiol levels	0.0001	0.0001			1.225	0.228	− 0.0001	0.0001
	Progesterone levels	− 0.0001	0.0001			− 0.556	0.581	− 0.001	0.0001
	LGCs normal morphology	− 0.420	0.361			− 1.163	0.252	− 1.151	0.311
	LGCs vitality	− 0.260	0.349			− 0.745	0.461	− 0.966	0.446
	*Probability distribution: normal, link function: identity*								
The size of the polar body I (µm)	Intercept	5.788	0.352			16.442	< 0.0001	5.094	6.482
	mtDNA/gDNA ratio	− 0.041	0.020			− 2.029	0.044	− 0.082	− 0.001
	Estradiol levels	− 0.0001	0.0001			− 1.087	0.279	− 0.0001	0.0001
	Progesterone levels	0.0001	0.0001			1.343	0.181	− 0.0001	0.0001
	LGCs normal morphology	− 0.001	0.003			− 0.313	0.755	− 0.007	0.005
	LGCs vitality	0.004	0.004			1.029	0.305	− 0.003	0.011
	*Probability distribution: gamma, link function: log*								
% of oocytes with fragmented polar body	Intercept	4.855	0.547			8.883	< 0.0001	3.718	5.992
	mtDNA/gDNA ratio	0.026	0.034			0.741	0.467	− 0.046	0.097
	Estradiol levels	0.0001	0.0001			1.685	0.107	− 0.0001	0.0001
	Progesterone levels	0.0001	0.0001			0.159	0.875	− 0.0001	0.0001
	LGCs normal morphology	− 0.008	0.007			− 1.069	0.297	− 0.023	0.007
	LGCs vitality	− 0.013	0.006			− 2.283	0.033	− 0.025	− 0.001
	*Probability distribution: gamma, link function: log*								
% of oocytes with small vacuoles	Intercept	1.346		20.774		0.065	0.949	− 40.786	43.478
	mtDNA/gDNA ratio	1.285		1.297		0.991	0.328	− 1.346	3.916
	Estradiol levels	0.0001		0.0001		1.796	0.081	− 0.0001	0.0001
	Progesterone levels	0.0001		0.0001		1.603	0.118	− 0.0001	0.0001
	LGCs normal morphology	− 0.332		0.189		− 1.756	0.088	− 0.715	0.051
	LGCs vitality	0.103		0.182		0.569	0.573	− 0.265	0.472
	*Probability distribution: normal, link function: identity*								
% of oocytes with small inclusions	Intercept	99.279	44.202		2.246		0.031	9.795	188.762
	mtDNA/gDNA ratio	0.017	2.719		0.006		0.995	− 5.488	5.522
	Estradiol levels	0.0001	0.0001		0.020		0.984	− 0.0001	0.0001
	Progesterone levels	0.001	0.0001		2.554		0.015	0.0001	0.001
	LGCs normal morphology	− 0.131	0.402		− 0.327		0.746	− 0.946	0.683
	LGCs vitality	− 0.522	0.380		− 1.373		0.178	− 1.293	0.248
	*Probability distribution: normal, link function: identity*								
% of oocytes with central granularity	Intercept	21.392	26.335		0.812		0.422	− 31.919	74.704
	mtDNA/gDNA ratio	− 1.012	1.620		− 0.625		0.536	− 4.292	2.267
	Estradiol levels	− 0.0001	0.0001		− 0.780		0.440	− 0.0001	0.0001
	Progesterone levels	− 0.0001	0.0001		− 0.149		0.882	− 0.0001	0.0001
	LGCs normal morphology	0.130	0.240		0.542		0.591	− 0.355	0.615
	LGCs vitality	− 0.185	0.227		− 0.816		0.420	− 0.644	0.274
	*Probability distribution: normal, link function: identity*								
Maturation rate (%)	Intercept	99.987	32.616		3.063		0.004	34.028	165.765
	mtDNA/gDNA ratio	− 1.146	1.992		− 0.575		0.568	− 5.169	2.878
	Estradiol levels	0.0001	0.0001		0.547		0.587	− 0.0001	0.0001
	Progesterone levels	− 0.0001	0.0001		− 1.599		0.117	− 0.001	0.0001
	LGCs normal morphology	− 0.122	0.296		− 0.413		0.682	− 0.719	0.475
	LGCs vitality	0.085	0.281		− 0.301		0.765	− 0.652	0.483
	*Probability distribution: normal, link function: identity*								
The number of cumulus-oocyte complexes recovered	Intercept	10.641	8.683		1.225		.227	− 6.896	28.177
	mtDNA/gDNA ratio	99.975	5304.129		0.019		0.985	− 10,611	10,811.881
	Estradiol levels	5.988E − 07	0.0001		0.444		0.659	0.0001	0.0001
	Progesterone levels	− 9.669E − 05	0.0001		− 1.841		0.073	0.0001	0.0001
	LGCs normal morphology	0.026	0.079		0.336		0.739	-0.133	0.185
	LGCs vitality	0.031	0.075		0.412		0.682	-0.120	0.182
	*Probability distribution: normal, link function: identity*								

*LGCs* luteal granulosa cells

#### PB I size

The results of the GLMM indicated that the mtDNA/gDNA ratio in LGCs may significantly affect the size of the PBI (*p* = 0.04) (Table 2). The coefficient of − 0.041 was exponentiated to obtain a value of approximately 0.960. This indicates that a one-unit increase in the mtDNA/gDNA ratio in LGCs is expected to lead to an approximately 4.0% decrease in PBI size (µm), assuming all other variables remain constant. This suggests that alterations in the mtDNA/gDNA ratio in LGCs could potentially be associated with a significant reduction in PBI size.

#### PBI fragmentation

The vitality of LGCs was observed to have a significant influence on the percentage of oocytes with fragmented PBI (*p* = 0.03) (Table 2), with a coefficient of − 0.013. When this coefficient is exponentiated, it results in a value of approximately 0.987. This implies that a 1.3% decrease in the percentage of oocytes with fragmented PBI is expected for every 1% increase in the percentage of LGC vitality, assuming all other variables remain constant.

#### Presence of small vacuoles

The GLMM results revealed that the percentage of oocytes with small vacuoles was not affected by the assessed factors including mtDNA/gDNA ratio in LGCs, estradiol and progesterone levels, the percentage of LGCs with normal morphology, and the percentage of viable LGCs (Table 2).

#### Presence of small inclusions

The results of the present study indicate a significant association between progesterone levels and the percentage of oocytes with small inclusions (*p* = 0.015) (Table 2). The coefficient of 0.001 suggests that for every one-unit (ng/mL) increase in progesterone level, there is a corresponding 0.1% increase in the percentage of oocytes with small inclusions, assuming all other variables remain constant.

#### Presence of central granularity

The GLMM analysis indicated that the percentage of oocytes with central granularity was not affected by the analyzed factors (Table 2).

#### Maturation rate and the number of cumulus-oocyte complexes recovered

The GLMM results revealed that the assessed independent variables (Table 2), including the mtDNA/gDNA ratio in luteal granulosa cells, estradiol and progesterone levels, the percentage of luteal granulosa cells with normal morphology, and the vitality of luteal granulosa cells, were not associated with the maturation rate and the number of cumulus-oocyte complexes recovered.

### Identifying associations between mtDNA content, vitality of LGCs, progesterone levels, and mature oocyte dysmorphisms through CATPCA analysis

Since statistically significant relationships were observed between mtDNA/gDNA ratio, progesterone levels, LGCs vitality, PVS size, PVS debris, PBI size, PBI fragmentation, and small inclusions, a CATPCA was performed to comprehensively analyze these relationships. Based on the results obtained from the CATPCA analysis, the analysis revealed the existence of three distinct dimensions (Fig. 2D, E). Each dimension appeared to consist of specific variables along with their corresponding factor loadings, as indicated under dimensions 1, 2, and 3.

In dimension 1, the variables mtDNA/gDNA ratio, LGCs vitality, PVS debris, and small cytoplasmic inclusion exhibited factor loadings of − 0.713, 0.707, − 0.579, and − 0.445, respectively. This suggests a strong association between these variables within this dimension. The negative factor loadings indicate that a decrease in LGCs viability tends to increase mtDNA/gDNA ratio, debris in the PVS, and small inclusions in the cytoplasm.

In dimension 2, the variable PVS size exhibited a factor loading of 0.783 (Fig. 2E). The PVS size was positively associated with the presence of debris in the PVS and negatively associated with PBI size (Fig. 2D).

In dimension 3, the variables fragmented PBI, PBI size, and progesterone level exhibited factor loadings of − 0.650, 0.455, and 0.605, respectively. The negative loading for fragmented PBI and the positive loadings for PBI size and progesterone level indicate an inverse relationship between these variables within this dimension.

### The impact of oocyte dysmorphisms on embryo morphokinetics

#### PVS size and debris presence

The PVS size was found to be correlated with the time to three cells (t3) (*R* =  − 0.018, *p* = 0.016) and time to five cells (t5) (*R* = 0.16, *p* = 0.023) (Supplementary Table 1). Furthermore, the presence of debris in the PVS was associated with a shorter time to t2 (*p* = 0.026) (Supplementary Table 2).

#### PBI size and fragmentation

The PBI size was positively correlated with the time to start compaction (*R* = 0.18, *p* = 0.034) (Supplementary Table 1). Additionally, a higher percentage of fragmented PBI was found in non-fertilized oocytes compared to fertilized oocytes (38.30% vs. 24%; *p* < 0.05) (Supplementary Fig. 1).

#### Small cytoplasmic inclusions

The presence of small cytoplasmic inclusions delayed the t2 (*p* = 0.024) and t7 (*p* = 0.003) in embryos derived from these oocytes compared to oocytes without small cytoplasmic inclusions (Supplementary Table 2).

#### Small cytoplasmic vacuoles

The presence of small cytoplasmic vacuoles was associated with a delay in the tPNf (*p* = 0.03) and an acceleration in the t2 (*p* < 0.01), t3 (*p* = 0.006), t4 (*p* = 0.03), and t5 (*p* = 0.02) compared to embryos derived from oocytes without small cytoplasmic vacuoles (Supplementary Table 2).

#### Central cytoplasmic granularity

The presence of central cytoplasmic granularity was associated with a delay in the tPNf (*p* = 0.03), t2 (*p* = 0.021), and the start of compaction time (*p* = 0.01) compared to embryos derived from oocytes without central cytoplasmic granularity (Supplementary Table 2).

### The impact of LGC parameters and oocyte dysmorphisms on live birth/single embryo transfer

There were no statistically significant differences between women who achieved live birth/single embryo transfer and those who did not, concerning mtDNA/gDNA ratio in LGCs (*p* = 0.56), normal LGC morphology (*p* = 0.18), LGC vitality (*p* = 0.22), estradiol level (*p* = 0.96), progesterone level (*p* = 0.4), maturation rate (*p* = 0.18), percentage of expanded COC (*p* = 0.63), percentage of oocytes with debris in the PVS (*p* = 0.57), percentage of oocytes with fragmented PBI (*p* = 0.52), percentage of oocytes with small vacuoles (*p* = 0.72), percentage of oocytes with small cytoplasmic inclusions (*p* = 0.48), and percentage of oocytes with central cytoplasmic granularity (*p* = 0.49) (Supplementary Table 3).

## Discussion

### Principal findings

The current study indicated that certain oocyte dysmorphisms were more likely to occur together, such as a decrease in PBI size, fragmentation of the PBI, the presence of debris in the PVS, an increase in the size of the PVS, and the presence of small cytoplasmic inclusions. Thereafter, this study analyzed the association of various follicular factors on oocyte dysmorphisms. We found that mtDNA LGC content, LGC vitality, and progesterone levels in the follicular fluid were associated with the size and fragmentation of the PBI, the size and presence of debris in the PVS, and the presence of small cytoplasmic inclusions. Additionally, this study showed that these oocyte dysmorphisms, in addition to central cytoplasmic granularity and small cytoplasmic vacuoles, were associated with preimplantation embryo morphokinetic parameters.

### Discussion of findings in the literature

In the present study, we observed an association among various oocyte dysmorphisms linked to oocyte over-maturation and aging (PBI fragmentation, presence of debris in the PVS, size of the PVS, and presence of small cytoplasmic inclusions). However, no association was found between these parameters and central cytoplasmic granularity; the latter may be indicative of cytoplasmic immaturity. Oocyte maturation takes place during the luteinizing hormone (LH) surge prior to ovulation and involves nuclear and cytoplasmic maturation. It entails nuclear maturation that results in the extrusion of the PBI and meiotic arrest at metaphase II (MII) in the oocyte [54, 55] as well as cytoplasmic maturation that results in the reorganization of organelles and molecular maturation. Molecular maturation itself is responsible for the storage of mRNAs, proteins, lipids, and transcription factors necessary for fertilization and early preimplantation embryo development [56–58]. However, the ability of oocytes to undergo successful meiotic maturation to the MII stage does not guarantee that cytoplasmic maturation has also been completed successfully [59]. One example of a sign of oocyte cytoplasmic immaturity is the presence of central cytoplasmic granulation, which is an abnormal accumulation of mitochondria and vesicles in the cytoplasm of an oocyte [60, 61]. In contrast, over-maturation of oocytes may lead to PBI fragmentation, an increase in the size of the PVS, and the presence of debris in the PVS [6, 62, 63]. Furthermore, cellular aging can lead to the accumulation of metabolic waste materials in the oocyte. This accumulation can lead to the appearance of small cytoplasmic circular inclusions (refractile bodies), and subsequently may impair embryo development. These inclusions form due to oxidative stress and changes in lipid metabolism [5].

Analysis through GLMM and CATPCA, revealed that certain oocyte dysmorphisms are associated with features encountered in over-mature and aged oocytes, including a higher percentage of oocytes with fragmented PBI, the presence of debris in the perivitelline space (PVS), and a higher percentage of oocytes with small inclusions were observed to increase as the percentage of luteal granulosa cell (LGC) vitality decreased. Assessing the characteristics of LGCs as well as hormone levels in the follicular fluid in conjunction with oocyte quality is important due to the pivotal role of granulosa cells in steroid synthesis and communication with the oocyte. Successful follicular development is crucial for producing competent oocytes, and this process relies on effective communication between follicular cells [64]. The follicular fluid that surrounds the oocyte consists of signaling molecules that circulate between granulosa cells and the oocyte during folliculogenesis and oocyte maturation [65]. Supply of nutrients and metabolites from granulosa cells to the oocyte is achieved by gap junctions, while granulosa cells also secrete paracrine signals that regulate the oocyte [66]. Gap junctions between the cumulus cells and oocyte, between the mural granulosa cells, and between the mural and cumulus cells are required for transmission of molecules. Moreover, molecules produced by the mural granulosa cells may travel to the oocyte through the follicular fluid [67]. Thus, the lack of granulosa cells deprives the oocyte of necessary nutrients, making it more susceptible to cell death [68].

In this context, our study revealed a significant negative correlation between the percentage of vital LGCs and the mtDNA/gDNA ratio in LGCs. Indeed, mitochondria play a pivotal role in the normal functioning of granulosa cells. For instance, mitochondria provide energy, maintain redox balance, facilitate metabolism, and calcium signaling for different cellular activities. Particularly, cumulus cells are highly glycolytic, generating pyruvate that can diffuse into the oocyte to produce ATP through mitochondria. This metabolic coordination is crucial for oocyte maturation, to preserve spindle/chromosome integrity, and prevent the deterioration of oocyte quality [69, 70]. In parallel, as shown from LGCs of young women, mitochondria are associated with a physiologically appropriate level of energy production [21, 71]. However, under certain conditions (such as aging, PCOS, endometriomas, premature ovarian insufficiency, impaired glycolytic metabolism, and psychological stress), granulosa cells and oocytes may not have adequate energy substrates [21, 22, 31, 72–74]. This can trigger compensatory responses in mitochondrial dynamics and function. These responses may include changes in mitochondrial morphology to maintain high energy production. Additionally, an increase in mitochondrial biogenesis and changes in the expression of oxidative phosphorylation (OXPHOS) components may occur to meet the energy demands [21]. However, this compensatory response cannot sustain the energy supply over an extended period of time. Instead, it leads to the production of more reactive oxygen species (ROS) due to oxidative phosphorylation (OXPHOS) dysfunction. This creates a vicious cycle between oxidative stress and mitochondrial damage, ultimately harming the oocyte and leading to granulosa cell death [21, 22, 31, 68, 72–75].

Interestingly, in this study, the results of GLMM and CATPCA revealed associations between factors in LGCs (mtDNA content and vitality) and oocyte dysmorphisms related to oocyte aging and over-maturation (including PBI size, PBI integrity, presence of debris in the perivitelline space (PVS), and presence of small cytoplasmic inclusions). This could be supported by studies showing that optimal growth and maturation of oocytes requires a well-balanced and timed energy metabolism. Meiotic defects, organelle dysfunction, and epigenetic alterations have been linked to decreased levels of acid transport proteins, increased glucose/lipid content, and elevated reactive oxygen species in oocytes [9, 76]. Nonetheless, there is strong evidence in the literature that the quality, quantity, and distribution of oocyte mitochondria are essential in providing the amount of ATP needed to undergo cytoplasmic maturation, fertilization, and acquiring developmental competence [77–79]. Furthermore, fragmented polar bodies likely reflect cytoplasmic incompetence [80]. However, in order to evaluate whether the observed outcomes in terms of PBI size and integrity are caused by the mtDNA/gDNA ratio in LGCs, the oocyte constitution in terms of mitochondrial quantity and distribution would also need to be taken into consideration.

Our study also suggested a potential link between elevated progesterone levels and oocyte dysmorphisms related to aging and over-maturation, such as compromised PBI integrity and an increase in small cytoplasmic inclusions. Beyond the cellular environment within the follicle, the development of the oocyte is heavily influenced by the biochemical environment of the follicular fluid [65] [84]. The latter serves as a crucial mediator in facilitating communication between cells within the antral follicle, and also functions to transport nutrients to the oocyte [84]. Following the LH surge, preovulatory follicular fluid is dominated by progesterone, a steroid hormone [84]. Progesterone has the potential to regulate various biological processes within the ovarian tissue and feto-maternal unit, such as the resumption of meiosis, fertilization, embryonic development, and implantation [84]. The level of progesterone may simply indicate the quality of the follicle and the oocyte it contains, but the exact mechanism by which progesterone affects oocytes is not fully comprehended [84]. Previous studies have shown links of progesterone with fertilization and oocyte maturation [84, 85].

To investigate the impact of oocyte dysmorphisms on embryo development, we analyzed data on embryo morphokinetics collected from the time-lapse incubator (TLI). Our findings demonstrate that oocyte dysmorphisms such as debris in the PVS, small cytoplasmic inclusions, cytoplasmic vacuoles, and central cytoplasmic granularity are associated with the time it takes to reach the two-cell stage (t2). Previous studies have shown that t2 is linked to embryo blastulation and successful implantation [48]. Additionally, our results indicate that the time to reach the three-cell stage (t3) can be influenced by the size of the PVS and the presence of small cytoplasmic vacuoles, which are associated with embryo implantation potential [48]. Moreover, the presence of small cytoplasmic vacuoles can impact the time it takes to reach the four-cell stage (t4), which is also associated with embryo blastulation and successful implantation [48]. It is worth mentioning that vacuolization is one of the features associated with degeneration of the oocyte [86] and has shown to be associated with impaired early embryo development [45]. Our research further indicates that the time it takes to reach the five-cell (t5) stage is associated with the size of the PVS and small cytoplasmic inclusions. Additionally, the time it takes to reach the seven-cell (t7) stage is associated with central cytoplasmic granularity. Previous studies have demonstrated that t5, t7, and t8 are linked to blastulation and implantation rates [48]. Notably, our findings suggest that PBI size is associated with the time of morula starting compaction, which has been previously associated with implantation rate [48]. Furthermore, our results showed that the PBI integrity might affect the fertilization potential of injected oocytes.

Our results did not show any correlation between the assessed parameters, including granulosa cell characteristics, hormone levels, mtDNA/gDNA ratio, and the rate of specific dysmorphisms per patient oocyte cohort, with live birth following the transfer of a single embryo. The limited sample size and preferential selection of oocytes without dysmorphisms precluded clinical outcome comparisons for transferred embryos. In the literature, previous studies have examined prevalence of specific oocyte dysmorphisms, with clinical outcomes with conflicting outcomes. An association between oocyte dysmorphisms and clinical outcomes was demonstrated when more than 50% of dysmorphic oocytes were present in repeated cycles with the same dimorphism, despite 33% of transferred embryos being morphologically normal [89]. When it comes to specific oocyte dysmorphism, high prevalence of central granulation has been shown to be associated with reduced clinical outcomes [90], while no significance in clinical outcomes after transfer of embryos deriving from oocytes with central granulation has been shown [61]. It has to be noted that embryos derived from dysmorphic oocytes have been shown to exhibit good embryonic development and embryo quality [91].

### Limitations

This study is an observational investigation conducted through a retrospective analysis of data. Due to the retrospective nature and conditions of clinical practice, follicular fluid from individual follicles and direct links to individual oocyte characteristics could not be assessed. Furthermore, information about the total number and diameter of follicles aspirated was not available. Retrospective manual annotations of oocytes were carried out, which could potentially increase the risk of bias. Even though strict exclusion criteria were followed, the population consisted of 47% smokers and 10% patients with PCOS and the outcomes therefore may not be universally applicable to the infertile population seeking fertility treatment. Furthermore, the small sample size when assessing pregnancy outcomes, accompanied by the fact that oocytes with dysmorphisms as assessed via the embryo viewer software were deselected for ET, is a limiting factor. The potential for improved generalization of the findings could be substantiated through the implementation of a prospective study.

In terms of methodological limitations, the study employed LGCs obtained following transvaginal oocyte retrieval, a process that involves penetrating vascularized structures. The resulting cell population is heterogeneous, comprising LGCs mixed with various cell types due to contamination. The use of density gradient as a method of purification might have led to an overestimation of cell vitality. However, caution is advised when interpreting studies lacking purified LGCs, as molecular and cell culture findings may be influenced. To ascertain the specific role of LGCs in follicle development and ovarian function, isolation techniques are necessary to obtain a pure or nearly pure cell population [83]. Future investigations employing LGCs purification techniques could be based on the recognition of specific cell markers. These approaches may yield increased purity at the cost of a potentially lower LGCs recovery rate when compared to the cell aggregation method utilized in our study [83].

Data on cell vitality was acquired through the trypan blue exclusion test. While this assay provides valuable insights into cellular vitality, a necessity arises for more comprehensive and profound analyses to further elucidate the intricate aspects of granulosa cell vitality and metabolic dynamics. Subsequent investigations could yield advantages by integrating an array of complementary techniques to enrich our comprehension of granulosa cell biology. Specifically, the integration of cell vitality fluorescent assays, such as propidium iodide or ethidium bromide, would facilitate the visualization and quantification of cell viability based on membrane integrity [81]. Moreover, employing mitochondrial fluorescent probes could assist in establishing a direct correlation between metabolic activity and fluorescence intensity [82].

### Perspectives and broader implications

Even though time-lapse systems have been routinely implemented in clinical settings, their efficacy in terms of improving clinical outcomes is still questioned [27, 28]. Incorporation of an automatic or artificial intelligence-driven protocol that evaluates oocyte morphology through analysis of hundreds of thousands of existing time-lapse video recordings with known implantation results and matching them with oocyte morphology data could provide insights that could facilitate improved embryo selection. This technology may determine if there is a proven correlation between oocyte morphology and pregnancy outcome, which could enhance the value of embryo morphokinetic data. Previous research has established linkage of oocyte morphology with cleavage patterns in human embryos, such as uneven blastomeres, reverse cleavage, and direct and arbitrary cleavages [5]. In addition, future investigations could broaden their scope by examining mtDNA/gDNA ratio within luteal granulosa cells (LGCs) from each follicle, thereby exploring potential correlations with oocyte outcomes. Furthermore, assessing the levels of steroids in the follicular fluid of each follicle could provide valuable insights into the intricate interplay between the hormonal milieu and oocyte quality. Additionally, further studies could explore the correlation between serum estradiol and progesterone levels and the assessed parameters in this study, including the presence of debris in the PVS, PVS size, and the percentage of mature oocytes with fragmented PBI. Altogether, these indicators may enhance our understanding of both the optimal management of ovarian stimulation and the ideal timing for triggering ovulation. Expanding the sample size and thoroughly assessing the implications for pregnancy outcomes would further enhance our understanding of these complex relationships.

## Conclusion

In conclusion, this study suggests that LGC parameters, including the mtDNA/gDNA ratio, vitality, and follicular fluid progesterone levels, are important contributors to oocyte quality. Upon verification, evaluation of these parameters could be embedded in clinical practice for categorization of oocytes based on quality characteristics for use in assisted reproductive technologies (ART). The association of LGC vitality, mtDNA content, and progesterone levels with several oocyte features and the relationship between these features and preimplantation embryo development can enable us to understand the potential causes of observed dysmorphisms in cohorts of oocytes collected from patients and the subsequent compromised development of embryos. This comprehension can be applied in patient counseling to effectively manage expectations. Ongoing research in this area can focus on identifying lifestyle interventions that may positively impact the metabolism and physiology of the body systems in general, with a specific emphasis on the ovary.

### Supplementary Information

Below is the link to the electronic supplementary material.

Supplementary file1 (DOCX 16 KB)

Supplementary file2 (DOCX 33 KB)

Supplementary file3 (DOCX 19 KB)

Supplementary file4 (PPTX 62 KB)

## Data Availability

The dataset supporting the conclusions of this article is included within the article.

## References

[1] Wyns C, Bergh C, Calhaz-Jorge C, De Geyter C, Kupka MS, Motrenko T, et al. ART in Europe, 2016: results generated from European registries by ESHRE†. Hum Reprod Open. 2020;2020:hoaa032. 10.1093/hropen/hoaa032 10.1093/hropen/hoaa032 PMC7394132 32760812

[2] Zagadailov P, Hsu A, Seifer DB, Stern JE. Differences in utilization of Intracytoplasmic sperm injection (ICSI) within human services (HHS) regions and metropolitan megaregions in the U.S. Reprod Biol Endocrinol. 2017;15:45. 10.1186/s12958-017-0263-4 10.1186/s12958-017-0263-4 PMC5469007 28606175

[3] Setti AS, Figueira RCS, Braga DPAF, Colturato SS, Iaconelli A, Borges E (2011). Relationship between oocyte abnormal morphology and intracytoplasmic sperm injection outcomes: a meta-analysis. Eur J Obstet Gynecol Reprod Biol.

[4] Bartolacci A, Intra G, Coticchio G, dell’Aquila M, Patria G, Borini A (2022). Does morphological assessment predict oocyte developmental competence? A systematic review and proposed score. J Assist Reprod Genet.

[5] Nikiforov D, Grøndahl ML, Hreinsson J, Andersen CY (2022). Human oocyte morphology and outcomes of infertility treatment: a systematic review. Reprod Sci.

[6] Magli MC, Jones GM, Lundin K, van den Abbeel E (2012). Atlas of human embryology: from oocytes to preimplantation embryos. Preface Hum Reprod.

[7] Uyar A, Torrealday S, Seli E (2013). Cumulus and granulosa cell markers of oocyte and embryo quality. Fertil Steril Am Soc Reproductive Med.

[8] Dompe C, Kulus M, Stefańska K, Kranc W, Chermuła B, Bryl R, et al. Human granulosa cells—stemness properties, molecular cross-talk and follicular angiogenesis. Cells. 2021;10. 10.3390/cells10061396 PMC8229878 34198768

[9] Sutton-McDowall ML, Gilchrist RB, Thompson JG (2010). The pivotal role of glucose metabolism in determining oocyte developmental competence. Reproduction.

[10] Sutton-McDowall ML, Mottershead DG, Gardner DK, Gilchrist RB, Thompson JG (2012). Metabolic differences in bovine cumulus-oocyte complexes matured in vitro in the presence or absence of follicle-stimulating hormone and bone morphogenetic protein 151. Biol Reprod.

[11] Turathum B, Gao EM, Chian RC (2021). The function of cumulus cells in oocyte growth and maturation and in subsequent ovulation and fertilization. Cells.

[12] Richani D, Gilchrist RB (2018). The epidermal growth factor network: role in oocyte growth, maturation and developmental competence. Hum Reprod Update.

[13] Tesarik JAN, Biology R, Hospital A, Mbdicale R, Biology M, Faculty G (1995). Oocytes : relationship to oocyte developmental. J Clin Endocrinol Metab.

[14] Tesarik J, Mendoza C (1997). Direct non-genomic effects of follicular steroids on maturing human oocytes: oestrogen versus androgen antagonism. Hum Reprod Update.

[15] Hasegawa J, Yanaihara A, Iwasaki S, Otsuka Y, Negishi M, Akahane T (2005). Reduction of progesterone receptor expression in human cumulus cells at the time of oocyte collection during IVF is associated with good embryo quality. Hum Reprod.

[16] Hu J, Zhang Z, Shen W-J, Azhar S (2010). Cellular cholesterol delivery, intracellular processing and utilization for biosynthesis of steroid hormones. Nutr Metab (Lond).

[17] Dai Q, Likes CE, Luz AL, Mao L, Yeh JS, Wei Z (2019). A mitochondrial progesterone receptor increases cardiac beta-oxidation and remodeling. J Endocr Soc.

[18] Behera MA, Dai Q, Garde R, Saner C, Jungheim E, Price TM (2009). Progesterone stimulates mitochondrial activity with subsequent inhibition of apoptosis in MCF-10A benign breast epithelial cells. Am J Physiol Metab. Am Physiol Soc.

[19] Klinge CM. Estrogenic control of mitochondrial function. Redox Biol . Elsevier B.V.; 2020;31:101435. 10.1016/j.redox.2020.101435 10.1016/j.redox.2020.101435 PMC7212490 32001259

[20] Yager JD, Chen JQ. Mitochondrial estrogen receptors - new insights into specific functions. Trends Endocrinol Metab . Elsevier; 2007;18:89–91. 10.1016/j.tem.2007.02.006 10.1016/j.tem.2007.02.006 17324583

[21] Liu Y, Han M, Li X, Wang H, Ma M, Zhang S (2017). Age-related changes in the mitochondria of human mural granulosa cells. Hum Reprod.

[22] Zhang Q, Ren J, Wang F, Pan M, Cui L, Li M (2022). Mitochondrial and glucose metabolic dysfunctions in granulosa cells induce impaired oocytes of polycystic ovary syndrome through Sirtuin 3. Free Radic Biol Med Pergamon.

[23] Bhargava D, Urs S, Wu W, Komrskova K, Postlerova P, Lin Y, et al. Mitochondrial function in modulating human granulosa cell steroidogenesis and female fertility. Int J Mol Sci. 2020; 10.3390/ijms21103592 PMC7279321 32438750

[24] Lamas-toranzo I, Gonz L, Alvarez PB-, Gonz P (2023). The human cumulus cell transcriptome provides poor predictive value for embryo transfer outcome. Reprod Biomed Online.

[25] Mantovani C, Luz D, Gomes M, Broi D, Koopman LDO, Plaça JR, et al. Transcriptomic analysis of cumulus cells shows altered pathways in patients with minimal and mild endometriosis. Sci Rep . Nature Publishing Group UK; 2022;1–9. 10.1038/s41598-022-09386-4 10.1038/s41598-022-09386-4 PMC8986826 35388025

[26] Meseguer M, Rubio I, Cruz M, Basile N, Marcos J, Requena A (2012). Embryo incubation and selection in a time-lapse monitoring system improves pregnancy outcome compared with a standard incubator: a retrospective cohort study. Fertil Steril Elsevier.

[27] Bamford T, Barrie A, Montgomery S, Dhillon-Smith R, Campbell A, Easter C (2022). Morphological and morphokinetic associations with aneuploidy: a systematic review and meta-analysis. Hum Reprod Update.

[28] Meng Q, Xu Y, Zheng A, Li H, Ph D, Ding J (2023). Noninvasive embryo evaluation and selection by time-lapse monitoring vs. conventional morphologic assessment in women undergoing in vitro fertilization / intracytoplasmic sperm injection : a single-center randomized controlled study. Fertil Steril. Am Soc Reproductive Med.

[29] Xin B, Hang M, Lei Z, Bo J. Neonatal outcomes of embryos cultured in a time ‑ lapse incubation system : an analysis of more than 15 , 000 fresh transfer cycles. Reprod Sci . Springer International Publishing; 2021;1–7. 10.1007/s43032-021-00714-z 10.1007/s43032-021-00714-z 34406638

[30] Tabibnejad N, Sheikhha MH, Ghasemi N, Fesahat F, Soleimani M, Aflatoonian A. Association between early embryo morphokinetics plus cumulus cell gene expression and assisted reproduction outcomes in polycystic ovary syndrome women. Reprod Biomed Online . Elsevier Ltd; 2019;38:139–51. 10.1016/j.rbmo.2018.10.010 10.1016/j.rbmo.2018.10.010 30593440

[31] Raad G, Tanios J, Kerbaj S, Mourad Y, Fakih F, Shamas F (2021). Stress management during the intracytoplasmic sperm injection cycle may slow down first embryo cleavage and accelerate embryo compaction: a pilot randomized controlled trial. Psychother Psychosom.

[32] Bakos HW, Henshaw RC, Mitchell M, Lane M. Paternal body mass index is associated with decreased blastocyst development and reduced live birth rates following assisted reproductive technology. Fertil Steril . Elsevier Ltd; 2011;95:1700–4. 10.1016/j.fertnstert.2010.11.044 10.1016/j.fertnstert.2010.11.044 21145051

[33] Bellver J, Mifsud A, Grau N, Privitera L, Meseguer M (2013). Similar morphokinetic patterns in embryos derived from obese and normoweight infertile women: a time-lapse study. Hum Reprod.

[34] Feil D, Henshaw RC, Lane M (2008). Day 4 embryo selection is equal to Day 5 using a new embryo scoring system validated in single embryo transfers. Hum Reprod.

[35] Yu L, Liu M, Wang Z, Liu T, Liu S, Wang B (2021). Correlation between steroid levels in follicular fluid and hormone synthesis related substances in its exosomes and embryo quality in patients with polycystic ovary syndrome. Reprod Biol Endocrinol.

[36] Floehr J, Dietzel E, Neulen J, Rösing B, Weissenborn U, Jahnen-Dechent W (2016). Association of high fetuin-B concentrations in serum with fertilization rate in IVF: a cross-sectional pilot study. Hum Reprod.

[37] Qu F, Wang F-F, Lu X-E, Dong M-Y, Sheng J-Z, Lv P-P (2010). Altered aquaporin expression in women with polycystic ovary syndrome: hyperandrogenism in follicular fluid inhibits aquaporin-9 in granulosa cells through the phosphatidylinositol 3-kinase pathway. Hum Reprod.

[38] Scalici E, Traver S, Molinari N, Mullet T, Monforte M, Vintejoux E (2014). Cell-free DNA in human follicular fluid as a biomarker of embryo quality. Hum Reprod.

[39] Raad G, Bazzi M, Tanios J, Mourad Y, Azouri J, Azouri J (2020). Optimization of the cell aggregates method for isolation and purification of human granulosa cells from follicular fluid. Int J Fertil Steril.

[40] Wells D, Kaur K, Grifo J, Glassner M, Taylor JC, Fragouli E (2014). Clinical utilisation of a rapid low-pass whole genome sequencing technique for the diagnosis of aneuploidy in human embryos prior to implantation. J Med Genet.

[41] Fragouli E, Spath K, Alfarawati S, Kaper F, Craig A, Michel CE (2015). Altered levels of mitochondrial DNA are associated with female age, aneuploidy, and provide an independent measure of embryonic implantation potential. PLoS Genet.

[42] Shenoy CC, Khan Z, Coddington CC, Stewart EA, Morbeck DE. Symmetry at the 4-cell stage is associated with embryo aneuploidy. Reprod Sci . Springer International Publishing; 2021;28:3473–9. 10.1007/s43032-021-00758-1 10.1007/s43032-021-00758-1 34664220

[43] Coello A, Meseguer M, Galán A, Alegre L, Remohí J, Cobo A (2017). Analysis of the morphological dynamics of blastocysts after vitrification/warming: defining new predictive variables of implantation. Fertil Steril.

[44] Park JK, Ahn S-Y, Seok SH, Park SY, Bang S, Eum JH, et al. Clinical usability of embryo development using a combined qualitative and quantitative approach in a single vitrified-warmed blastocyst transfer: assessment of pre-vitrified blastocyst diameter and post-warmed blastocyst re-expansion speed. J. Clin. Med. 2022. 10.3390/jcm11237085 PMC9736480 36498659

[45] Ebner T, Ph D, Moser M, Ph D, Sommergruber M. Occurrence and developmental consequences of vacuoles throughout preimplantation development. Fertil Steril. 2005;83. 10.1016/j.fertnstert.2005.02.009 15950630

[46] Balaban B, Brison D, Calderón G, Catt J, Conaghan J, Cowan L (2011). The Istanbul consensus workshop on embryo assessment: proceedings of an expert meeting. Hum Reprod.

[47] Lainas GT, Lainas TG, Sfontouris IA, Chatzimeletiou K, Venetis CA, Bosdou JK (2019). Is oocyte maturation rate associated with triptorelin dose used for triggering final oocyte maturation in patients at high risk for severe ovarian hyperstimulation syndrome. Hum Reprod.

[48] Apter S, Ebner T, Freour T, Guns Y, Kovacic B, Le Clef N (2020). Good practice recommendations for the use of time-lapse technology†. Hum Reprod Open.

[49] Meseguer M, Herrero J, Tejera A, Hilligsøe KM, Ramsing NB, Remohı J (2011). The use of morphokinetics as a predictor of embryo implantation. Hum Reprod.

[50] Marklund A, Eloranta S, Wikander I, Kitlinski ML, Lood M, Nedstrand E (2020). Efficacy and safety of controlled ovarian stimulation using GnRH antagonist protocols for emergency fertility preservation in young women with breast cancer - a prospective nationwide Swedish multicenter study. Hum Reprod.

[51] Geber S, Brandão AHF, Sampaio M (2012). Effects of estradiol and FSH on leptin levels in women with suppressed pituitary. Reprod Biol Endocrinol.

[52] Linting M, Van Der Kooij A (2012). Nonlinear principal components analysis with CATPCA: a tutorial. J Pers Assess.

[53] Vidal AF, Cruz AMP, Magalhães L, Pereira AL, Anaissi AKM, Alves NCF (2016). Hsa-miR-29c and hsa-miR-135b differential expression as potential biomarker of gastric carcinogenesis. World J Gastroenterol.

[54] PENG X-R, HSUEH AJW, LAPOLT PS, BJERSING L, NY T. Localization of luteinizing hormone receptor messenger ribonucleic acid expression in ovarian cell types during follicle development and ovulation*. Endocrinology . 1991;129:3200–7. 10.1210/endo-129-6-3200 10.1210/endo-129-6-3200 1954899

[55] Jamnongjit Stephen R MH. Oocyte maturation: the coming of age of a germ cell. Semin Reprod Med . 2005;23:234–41. Available from: http://www.thieme-connect.com/products/ejournals/abstract/10.1055/s-2005-872451 10.1055/s-2005-872451 PMC1482430 16059829

[56] Swain JE, Pool TB (2008). ART failure: oocyte contributions to unsuccessful fertilization. Hum Reprod Update.

[57] Mao L, Lou H, Lou Y, Wang N, Jin F. Behaviour of cytoplasmic organelles and cytoskeleton during oocyte maturation. Reprod Biomed Online . Reproductive Healthcare Ltd.; 2014;28:284–99. 10.1016/j.rbmo.2013.10.016 10.1016/j.rbmo.2013.10.016 24444815

[58] Reader KL, Stanton JAL, Juengel JL (2017). The role of oocyte organelles in determining developmental competence. Biology (Basel).

[59] Coticchio G, Dal Canto M, Mignini Renzini M, Guglielmo MC, Brambillasca F, Turchi D (2015). Oocyte maturation: gamete-somatic cells interactions, meiotic resumption, cytoskeletal dynamics and cytoplasmic reorganization. Hum Reprod Update.

[60] Sousa M, Cunha M, Silva J, Oliveira E, Pinho MJ, Almeida C (2016). Ultrastructural and cytogenetic analyses of mature human oocyte dysmorphisms with respect to clinical outcomes. J Assist Reprod Genet. J Ass Reproduction Gen.

[61] Kahraman S, Yakın K, Dönmez E, Şamlı H, Bahçe M, Cengiz G (2000). Relationship between granular cytoplasm of oocytes and pregnancy outcome following intracytoplasmic sperm injection. Hum Reprod.

[62] Ebner T (2009). Extracytoplasmic markers of human oocyte quality. J Mamm Ova Res.

[63] Miao YL, Kikuchi K, Sun QY, Schatten H (2009). Oocyte aging: cellular and molecular changes, developmental potential and reversal possibility. Hum Reprod Update.

[64] Huang Z, Wells D (2010). The human oocyte and cumulus cells relationship : new insights from the cumulus cell transcriptome.

[65] Revelli A, Piane LD, Casano S, Molinari E, Massobrio M, Rinaudo P (2009). Follicular fluid content and oocyte quality : from single biochemical markers to metabolomics. Reprod Biol Endocrinol.

[66] Eppig JJ. Reproduction: oocytes call, granulosa cells connect. Curr Biol . Elsevier Ltd; 2018;28:R354–6. 10.1016/j.cub.2018.03.005 10.1016/j.cub.2018.03.005 29689210

[67] Jaffe LA, Egbert JR. Regulation of mammalian oocyte meiosis by intercellular communication within the ovarian follicle. Rev Adv. 2017;1–24. 10.1146/annurev-physiol-022516-034102 PMC5305431 27860834

[68] Tatone C, Ph D, Amicarelli F, Ph D. The aging ovary — the poor granulosa cells. Fertil Steril . Elsevier Inc.; 2013;99:12–7. 10.1016/j.fertnstert.2012.11.029 10.1016/j.fertnstert.2012.11.029 23273984

[69] Adhikari D, Lee I, Yuen WS, Carroll J (2022). Oocyte mitochondria — key regulators of oocyte function and potential therapeutic targets for improving fertility. Biol Reprod.

[70] Richani D, Dunning KR, Thompson JG, Gilchrist RB (2021). Metabolic co-dependence of the oocyte and cumulus cells: essential role in determining oocyte developmental competence. Hum Reprod Update.

[71] Chien Y, Rosal K, Chung BC (2017). Function of CYP11A1 in the mitochondria. Mol Cell Endocrinol.

[72] Kauppila TES, Kauppila ohanna HK, Larsson N-G. Review mammalian mitochondria and aging : an update. Cell Metab 2017;25:1–15 10.1016/j.cmet.2016.09.017 28094012

[73] Mao J, Zhang J, Cai L, Cui Y, Liu J, Mao Y (2022). Elevated prohibitin 1 expression mitigates glucose metabolism defects in granulosa cells of infertile patients with endometriosis. Mol Hum Reprod.

[74] Li D, Wang X, Li G, Dang Y, Zhao S, Qin Y (2021). LncRNA ZNF674-AS1 regulates granulosa cell glycolysis and proliferation by interacting with ALDOA. Cell Death Discov.

[75] Wang J, Wu J, Zhang Y, Zhang J, Xu W, Wu C, Zhou P. Growth hormone protects against ovarian granulosa cell apoptosis: Alleviation oxidative stress and enhancement mitochondrial function. Reprod Biol. 2021;21(2):100504. 10.1016/j.repbio.2021.100504 10.1016/j.repbio.2021.100504 33839528

[76] Gu L, Liu H, Gu X, Boots C, Moley KH, Wang Q (2015). Metabolic control of oocyte development: linking maternal nutrition and reproductive outcomes. Cellular and molecular life sciences : CMLS.

[77] Kirillova A, Smitz JEJ, Sukhikh GT, Mazunin I (2021). The role of mitochondria in oocyte maturation. Cells.

[78] Liu S, Li Y, Gao X, Yan JH, Chen ZJ (2010). Changes in the distribution of mitochondria before and after in vitro maturation of human oocytes and the effect of in vitro maturation on mitochondria distribution. Fertil Steril.

[79] Babayev E, Seli E. Oocyte mitochondrial function and reproduction. Curr Opin Gynecol Obstet. 2015;27(3):175–81. 10.1097/GCO.0000000000000164. 10.1097/GCO.0000000000000164 PMC4590773 25719756

[80] Rose BI, Laky D (2013). Polar body fragmentation in IVM oocytes is associated with impaired fertilization and embryo development. J Assist Reprod Genet.

[81] Wu FJ, Wang YW, Luo CW. Human bone morphogenetic protein 8A promotes expansion and prevents apoptosis of cumulus cells in vitro. Mol Cell Endocrinol. 2021;522:111121. 10.1016/j.mce.2020.111121 10.1016/j.mce.2020.111121 33338549

[82] Lu X, Liu Y, Xu J, Cao X, Zhang D, Liu M, Liu S, Dong X, Shi H (2022). Mitochondrial dysfunction in cumulus cells is related to decreased reproductive capacity in advanced-age women. Fertil Steril.

[83] Ferrero H, Delgado-Rosas F, Garcia-Pascual CM, Monterde M, Zimmermann RC, Simón C, Pellicer A, Gómez R (2012). Efficiency and purity provided by the existing methods for the isolation of luteinized granulosa cells: a comparative study. Hum Reprod (Oxford, England).

[84] Nagy B, Poto L, Farkas N, Koppan M, Varnagy A, Kovacs K, Papp S, Bohonyi N, Bodis J (2019). Follicular fluid progesterone concentration is associated with fertilization outcome after IVF: a systematic review and meta-analysis. Reprod Biomed Online.

[85] Sun Z, Song J, Zhang X, Wang A, Guo Y, Yang Y, Wang X, Xu K, Deng J (2018). SWATHHM-Based Metabolomics of Follicular Fluid in Patients Shows That Progesterone Adversely Affects Oocyte Quality. Biomed Res Int.

[86] Sousa M, Cunha M, Silva J, Oliveira E, Pinho MJ, Almeida C, Sá R, da Silva JT, Oliveira C, Barros A (2016). Ultrastructural and cytogenetic analyses of mature human oocyte dysmorphisms with respect to clinical outcomes. J Assist Reprod Genet.

[87] Armstrong S, Bhide P, Jordan V, Pacey A, Farquhar C Time-lapse systems for embryo incubation and assessment in assisted reproduction. Cochrane Database Syst Rev. 2018;5(5):CD011320. 10.1002/14651858.CD011320.pub3 10.1002/14651858.CD011320.pub3 PMC6494546 29800485

[88] Kieslinger DC, Vergouw CG, Ramos L, Arends B, Curfs MHJM, Slappendel E, Kostelijk EH, Pieters MHEC, Consten D, Verhoeven MO, Besselink DE, Broekmans F, Cohlen BJ, Smeenk JMJ, Mastenbroek S, de Koning CH, van Kasteren YM, Moll E, van Disseldorp J, Brinkhuis EA, Lambalk CB (2023). Clinical outcomes of uninterrupted embryo culture with or without time-lapse-based embryo selection versus interrupted standard culture (SelecTIMO): a three-armed, multicentre, double-blind, randomised controlled trial. Lancet (London, England).

[89] Meriano JS, Alexis J, Visram-Zaver S, Cruz M, Casper RF (2001). Tracking of oocyte dysmorphisms for ICSI patients may prove relevant to the outcome in subsequent patient cycles. Hum Reprod.

[90] Merviel P, Cabry R, Chardon K, Haraux E, Scheffler F, Mansouri NB, Devaux A, Chahine H, Bach V, Copin H, Benkhalifa M (2017). Impact of oocytes with CLCG on ICSI outcomes and their potential relation to pesticide exposure. J Ovarian Res.

[91] Yu EJ, Ahn H, Lee JM, Jee BC, Kim SH (2015). Fertilization and embryo quality of mature oocytes with specific morphological abnormalities. Clin Exp Reprod Med Korea (South).

