# Supplementary material for: Mature oocyte dysmorphisms may be associated with progesterone levels, mitochondrial DNA content, and vitality in luteal granulosa cells
Source: J Assist Reprod Genet. 2024 Feb 16;41(3):795–813. doi: 10.1007/s10815-024-03053-5 (PMC10957819; doi:10.1007/s10815-024-03053-5)
Supplement: Supplementary file 1 — Supplementary file1 (DOCX 16 KB) [file 10815_2024_3053_MOESM1_ESM.docx]

**Supplementary table 1: Correlations between PVS size, PBI size and embryo morphokinetics**

|  | **PVS (µm)** | **PBI (µm)** |
| --- | --- | --- |
| **tPBII extrusion** | R = 0.11  p = 0.1 | R = -0.06  p = 0.4 |
| **tPNa** | R = 0.06  p = 0.33 | R = -0.07  p = 0.35 |
| **tPNf** | R = 0.06  p = 0.34 | R = 0.05  p = 0.43 |
| **t2** | R = 0.04  p = 0.53 | R = 0.075  p = 0.3 |
| **t3** | **R = -0.018**  **p = 0.016** | R = -0.08  p = 0.3 |
| **t4** | R = 0.10  p = 0.12 | R = 0.002  p = 0.9 |
| **t5** | **R = 0.16**  **p = 0.023** | R = 0.048  p = 0.58 |
| **t6** | R= 0.08  p = 0.27 | R= -0.012  p = 0.89 |
| **t7** | R = 0.1  p =0.24 | R = 0.078  p = 0.46 |
| **t8** | R = -0.03  p = 0.6 | R = 0.14  p = 0.1 |
| **t9** | R = 0.10  p = 0.17 | R = 0.14  p = 0.12 |
| **tstartcompaction** | R = 0.11  p = 0.12 | **R =** **0.18**  **p =** **0.034** |
| **tfullcompation** | R = -0.006  p =0.9 | R = -0.15  p = 0.15 |
