# Supplementary material for: Mature oocyte dysmorphisms may be associated with progesterone levels, mitochondrial DNA content, and vitality in luteal granulosa cells
Source: J Assist Reprod Genet. 2024 Feb 16;41(3):795–813. doi: 10.1007/s10815-024-03053-5 (PMC10957819; doi:10.1007/s10815-024-03053-5)
Supplement: Supplementary file 2 — Supplementary file2 (DOCX 33 KB) [file 10815_2024_3053_MOESM2_ESM.docx]

**Supplementary table 2: The impact of debris in the PVS, PBI fragmentation, and cytoplasmic dysmorphisms on embryo morphokineticks**

|  | | | | **p value** |
| --- | --- | --- | --- | --- |
| **tPBII extrusion** | Debris in the PVS | No | 2.9 (1.2-5.1) | 0.37 |
|  |  | Yes | 2.9 (0.7-15.5) |  |
|  | Fragmented PBI | No | 2.9 (1.2-6.2) | 0.47 |
|  |  | Yes | 3 (0.7-11.3) |  |
|  | Small cytoplasmic inclusions | No | 3 (0.7-5.3) | 0.46 |
|  |  | Yes | 2.9 (1.2 -15.5) |  |
|  | Small cytoplasmic vacuoles | No | 2.9 (1.2-15.5) | 0.16 |
|  |  | Yes | 2.4 (0.7-5.3) |  |
|  | Central cytoplasmic granularity | No | 2.9 (0.7-15.5) | 0.63 |
|  |  | Yes | 3.1 (1.8-5.1) |  |
| **tPNa** | Debris in the PVS | No | 7.45 (5.8-13.2) | 0.10 |
|  |  | Yes | 7.8 (5.4-20.5) |  |
|  | Fragmented PBI | No | 7.5 (5.4-20.5) | 0.10 |
|  |  | Yes | 7.9 (7.5-15.4) |  |
|  | Small cytoplasmic inclusions | No | 7.4 (6.1-10.1) | 0.29 |
|  |  | Yes | 7.7 (5.4-20.5) |  |
|  | Small cytoplasmic vacuoles | No | 7.7 (5.4-20.5) | 0.97 |
|  |  | Yes | 7.9 (6.7-9.1) |  |
|  | Central cytoplasmic granularity | No | 7.7 (5.4-20.5) | 0.60 |
|  |  | Yes | 8 (6.1-13.2) |  |
| tPNf | Debris in the PVS | No | 25.2 (17.9-37.6) | 0.08 |
|  |  | Yes | 24.1 (17.7-41.9) |  |
|  | Fragmented PBI | No | 25(17.7-40.6) | 0.16 |
|  |  | Yes | 23.8(18.1-41.9) |  |
|  | Small cytoplasmic inclusions | No | 25.3 (19.5-38.1) | 0.18 |
|  |  | Yes | 24.6 (17.7-41.9) |  |
|  | Small cytoplasmic vacuoles | No | 25 (17.9 – 41.9) | **0.01** |
|  |  | Yes | 21.4 (17.7 – 27.3) |  |
|  | Central cytoplasmic granularity | No | 24.4 (17.7 – 41.9) | **0.03** |
|  |  | Yes | 26.2 (20.4 –40.6) |  |
| t2 | Debris in the PVS | No | 28 (21-57) | **0.026** |
|  |  | Yes | 26.55 (0-44) |  |
|  | Fragmented PBI | No | 27.5 (0-57) | 0.323 |
|  |  | Yes | 26.8 (21-38) |  |
|  | Small cytoplasmic inclusions | No | 28.3 (22-57) | **0.024** |
|  |  | Yes | 27.3 (19.7-44) |  |
|  | Small cytoplasmic vacuoles | No | 27.6 (19.7-57) | **0.00** |
|  |  | Yes | 23.9 (20-30) |  |
|  | Central cytoplasmic granularity | No | 27.3 (19.7-57) | **0.021** |
|  |  | Yes | 29.95 (22-44) |  |
| t3 | Debris in the PVS | No | 35 (23.5-57.3) | 0.40 |
|  |  | Yes | 35.5 (23.2-61.8) |  |
|  | Fragmented PBI | No | 35.4 (23.5-61.8) | 0.77 |
|  |  | Yes | 35.9 (24.4-51) |  |
|  | Small cytoplasmic inclusions | No | 36.8 (24.9.2-57.3) | 0.13 |
|  |  | Yes | 35.7 (23.5-61.8) |  |
|  | Small cytoplasmic vacuoles | No | 36.5 (23.5-61.8) | **0.006** |
|  |  | Yes | 32.3 (24.6-35.3) |  |
|  | Central cytoplasmic granularity | No | 35.3 (24.4-61.8) | 0.101 |
|  |  | Yes | 35.45 (31.3-56) |  |
| t4 | Debris in the PVS | No | 38.9 (23.8-58.6) | 0.66 |
|  |  | Yes | 38.25 (23.8-68.8) |  |
|  | Fragmented PBI | No | 38.8 (23.8-68.8) | 0.31 |
|  |  | Yes | 38.9 (31.5-55.2) |  |
|  | Small cytoplasmic inclusions | No | 40.2 (30.7-58.6) | 0.77 |
|  |  | Yes | 38.3 (23.8-68.8) |  |
|  | Small cytoplasmic vacuoles | No | 39 (23.8-68.8) | **0.03** |
|  |  | Yes | 35.2 (30.5-48.9) |  |
|  | Central cytoplasmic granularity | No | 38.5 (26.1-68.8) | 0.13 |
|  |  | Yes | 42.2 (31-58.6) |  |
| t5 | Debris in the PVS | No | 49.2(27.3-87.2) | 0.47 |
|  |  | Yes | 48.8 (24.9-82.4) |  |
|  | Fragmented PBI | No | 49.7 (27.7-87.2) | 0.90 |
|  |  | Yes | 49.1 (24.9-82.4) |  |
|  | Small cytoplasmic inclusions | No | 51.4 (36.3-73.9) | 0.26 |
|  |  | Yes | 49.2 (24.9-87.2) |  |
|  | Small cytoplasmic vacuoles | No | 49.9 (24.9-87.2) | **0.02** |
|  |  | Yes | 46.2 (36.3-50.5) |  |
|  | Central cytoplasmic granularity | No | 49 (24.9-87.2) | 0.09 |
|  |  | Yes | 55.7 (34.3-66.9) |  |
| t6 | Debris in the PVS | No | 52.3 (33.1-87.3) | 0.45 |
|  |  | Yes | 52.2 (33.9-89.1) |  |
|  | Fragmented PBI | No | 52.5 (33.1-87.4) | 0.68 |
|  |  | Yes | 52.4 (33.9-89.1) |  |
|  | Small cytoplasmic inclusions | No | 52 (38.7-87.3) | 0.89 |
|  |  | Yes | 52.6 (33.1-89.1) |  |
|  | Small cytoplasmic vacuoles | No | 52.4 (33.1-89.1) | 0.51 |
|  |  | Yes | 51.3 (43.5-69.9) |  |
|  | Central cytoplasmic granularity | No | 52.1 (33.1-89.1) | 0.08 |
|  |  | Yes | 57.6 (42.1-87.4) |  |
| t7 | Debris in the PVS | No | 55.4 (33-83) | 0.73 |
|  |  | Yes | 55.5 (28-92) |  |
|  | Fragmented PBI | No | 55.05 (33-92) | 0.66 |
|  |  | Yes | 55.6 (41-82) |  |
|  | Small cytoplasmic inclusions | No | 64.7 (39-92) | **0.003** |
|  |  | Yes | 55.4 (33-88) |  |
|  | Small cytoplasmic vacuoles | No | 55.6 (33-92) | 0.36 |
|  |  | Yes | 60.75 (43-77) |  |
|  | Central cytoplasmic granularity | No | 55.85 (33-92) | 0.38 |
|  |  | Yes | 58.8 (51-76) |  |
| t8 | Debris in the PVS | No | 62.4 (44.3-94.7) | 0.30 |
|  |  | Yes | 60.8 (41.9-95) |  |
|  | Fragmented PBI | No | 69.9 (41.9-94.7) | 0.836 |
|  |  | Yes | 61.7 (45.1-95) |  |
|  | Small cytoplasmic inclusions | No | 64.9 (48.4-94.7) | 0.31 |
|  |  | Yes | 61.3 (41.9-95) |  |
|  | Small cytoplasmic vacuoles | No | 61.4 (41.9-95) | 0.98 |
|  |  | Yes | 59.9 (48.4-77.8) |  |
|  | Central cytoplasmic granularity | No | 60.7 (41.9-95) | 0.06 |
|  |  | Yes | 71.2 (56.8-77.2) |  |
| t9 | Debris in the PVS | No | 68.4 (48.2-93.7) | 0.81 |
|  |  | Yes | 68.9 (46.4-102.3) |  |
|  | Fragmented PBI | No | 67.8 (46.4-102.3) | 0.33 |
|  |  | Yes | 70.2 (46.4-92.6) |  |
|  | Small cytoplasmic inclusions | No | 75.1 (57.4-93.7) | 0.37 |
|  |  | Yes | 68.5 (46.4-102.3) |  |
|  | Small cytoplasmic vacuoles | No | 68.6 (46.4-102.3) | 0.94 |
|  |  | Yes | 67.9 (60-82.3) |  |
|  | Central cytoplasmic granularity | No | 67.9 (46.4-102.3) | **0.02** |
|  |  | Yes | 75.7 (62.1-93.7) |  |
| tstartcompaction | Debris in the PVS | No | 81 (60.9-105.2) | 0.58 |
|  |  | Yes | 79.7 (51.3-107.6) |  |
|  | Fragmented PBI | No | 79.6 (51.3-107.6) | 0.25 |
|  |  | Yes | 80 (66.9-105.2) |  |
|  | Small cytoplasmic inclusions | No | 80.1 (51.3-95.9) | 0.6 |
|  |  | Yes | 79.8 (54.9-107.6) |  |
|  | Small cytoplasmic vacuoles | No | 79.8 (51.3-107.6) | 0.93 |
|  |  | Yes | 80 (71.2-88.2) |  |
|  | Central cytoplasmic granularity | No | 79.7 (51.3-107.6) | **0.01** |
|  |  | Yes | 90.7 (76.2-97.2) |  |
| tfullcompaction | Debris in the PVS | No | 85.9 (74-113.2) | 0.11 |
|  |  | Yes | 80.7 (74-114.4) |  |
|  | Fragmented PBI | No | 82.8 (74-110) | 0.43 |
|  |  | Yes | 84.5 (74.113.2) |  |
|  | Small cytoplasmic inclusions | No | 89 (74-114.4) | 0.15 |
|  |  | Yes | 83.8 (74-113.2) |  |
|  | Small cytoplasmic vacuoles | No | 83 (74-113.2) | 0.88 |
|  |  | Yes | 82.3 (74-89.6) |  |
|  | Central cytoplasmic granularity | No | 82.3 (74-113.2) | 0.78 |
|  |  | Yes | 82.7 (74 – 95.6) |  |

Results are expressed as median (minimum – maximum). Statistical significance is considered when p<0.05.
