# Supplementary material for: Mature oocyte dysmorphisms may be associated with progesterone levels, mitochondrial DNA content, and vitality in luteal granulosa cells
Source: J Assist Reprod Genet. 2024 Feb 16;41(3):795–813. doi: 10.1007/s10815-024-03053-5 (PMC10957819; doi:10.1007/s10815-024-03053-5)
Supplement: Supplementary file 3 — Supplementary file3 (DOCX 19 KB) [file 10815_2024_3053_MOESM3_ESM.docx]

**Supplementary table 3:** **The impact of LGCs and oocyte morphology parameters on live birth/SET**

|  | **Live birth / SET** | **Mean** | **Std. Deviation** | **Std. Error Mean** | **p-value** |
| --- | --- | --- | --- | --- | --- |
| **mtDNA/gDNA ratio in LGCs** | **No** | 0.000368 | 0.000139 | 0.000024 | 0.562 |
|  | **Yes** | 0.000413 | 0.000213 | 0.000055 |  |
| **Normal morphology of LGCs (%)** | **No** | 78.62 | 10.58 | 1.81 | 0.18 |
|  | **Yes** | 74.50 | 10.66 | 2.85 |  |
| **LGCs vitality (%)** | **No** | 78.03 | 14.15 | 2.36 | 0.224 |
|  | **Yes** | 75.33 | 11.32 | 2.92 |  |
| **Estradiol (pg/ml)** | **No** | 1543783.33 | 651217.11 | 108536.18 | 0.967 |
|  | **Yes** | 1548126.67 | 572067.50 | 147707.19 |  |
| **Progesterone (ng/ml)** | **No** | 42186.11 | 16441.35 | 2740.22 | 0.408 |
|  | **Yes** | 47625.33 | 20982.78 | 5417.73 |  |
| **Percentage of expanded COC** | **No** | 49.55 | 36.27 | 6.05 | 0.631 |
|  | **Yes** | 54.68 | 36.82 | 9.51 |  |
| **Maturation rate (%)** | **No** | 69.41 | 20.45 | 3.41 | 0.188 |
|  | **Yes** | 78.52 | 19.29 | 4.98 |  |
| **Percentage of debris in the PVS** | **No** | 78.44 | 26.58 | 4.63 | 0.517 |
|  | **Yes** | 75.83 | 21.91 | 5.66 |  |
| **Percentage of oocytes with fragmented polar body** | **No** | 26.01 | 19.67 | 3.65 | 0.524 |
|  | **Yes** | 33.02 | 28.85 | 8.33 |  |
| **Percentage of oocytes with small cytoplasmic vacuoles** | **No** | 9.80 | 20.10 | 3.55 | 0.726 |
|  | **Yes** | 6.21 | 14.80 | 3.95 |  |
| **Percentage of oocytes with small cytoplasmic inclusions** | **No** | 77.56 | 31.40 | 5.38 | 0.484 |
|  | **Yes** | 84.52 | 24.65 | 6.59 |  |
| **Percentage of oocytes with central cytoplasmic granularity** | **No** | 6.17 | 14.36 | 2.46 | 0.492 |
|  | **Yes** | 8.04 | 18.38 | 4.91 |  |
