# Supplementary material for: Mature oocyte dysmorphisms may be associated with progesterone levels, mitochondrial DNA content, and vitality in luteal granulosa cells
Source: J Assist Reprod Genet. 2024 Feb 16;41(3):795–813. doi: 10.1007/s10815-024-03053-5 (PMC10957819; doi:10.1007/s10815-024-03053-5)
Supplement: Supplementary file 4 — Supplementary file4 (PPTX 62 KB) [file 10815_2024_3053_MOESM4_ESM.pptx]

## Slide 1
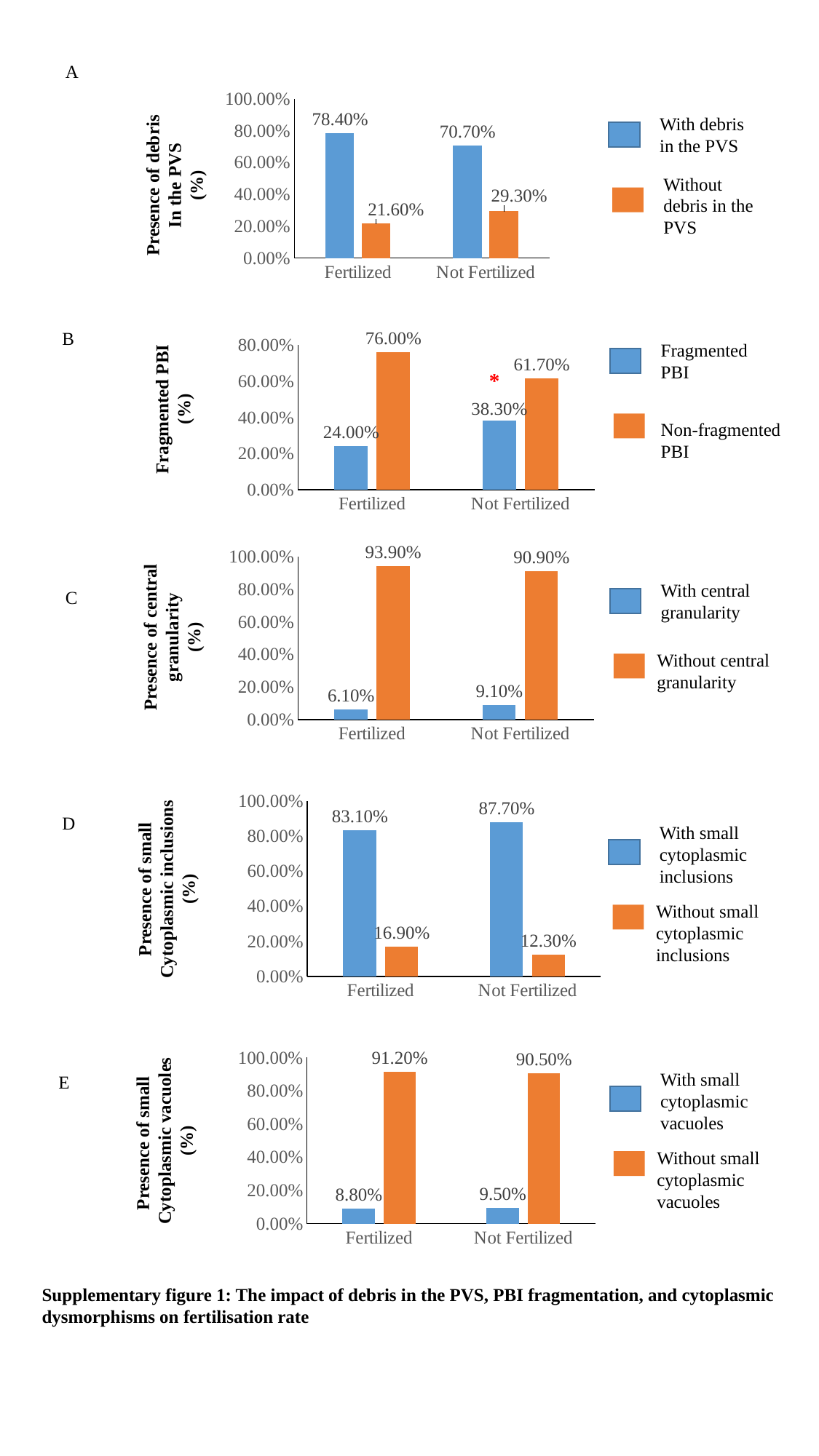

A
### Chart
| Category | PVS Debris | No PVS Debris |
|---|---|---|
| Fertilized | 0.784 | 0.216 |
| Not Fertilized | 0.707 | 0.293 |With debris in the PVS
Presence of debris
In the PVS
(%)
Without debris in the PVS
B
### Chart
| Category | Fragmented PB | Not Fragmented PB |
|---|---|---|
| Fertilized | 0.24 | 0.76 |
| Not Fertilized | 0.383 | 0.617 |Fragmented
PBI
Fragmented PBI
(%)
Non-fragmented
PBI
### Chart
| Category | Central granularity | No central granularity |
|---|---|---|
| Fertilized | 0.061 | 0.939 |
| Not Fertilized | 0.091 | 0.909 |With central granularity
C
Presence of central
granularity
(%)
Without central granularity
### Chart
| Category | Small Inclusions | No small Inclusions |
|---|---|---|
| Fertilized | 0.831 | 0.169 |
| Not Fertilized | 0.877 | 0.123 |D
With small cytoplasmic inclusions
Presence of small
Cytoplasmic inclusions
(%)
Without small cytoplasmic inclusions
### Chart
| Category | Small Vacuoles | No Small Vacuoles |
|---|---|---|
| Fertilized | 0.088 | 0.912 |
| Not Fertilized | 0.095 | 0.905 |With small cytoplasmic vacuoles
E
Presence of small
Cytoplasmic vacuoles
(%)
Without small cytoplasmic vacuoles
Supplementary figure 1: The impact of debris in the PVS, PBI fragmentation, and cytoplasmic dysmorphisms on fertilisation rate
